# High-throughput qPCR and 16S rRNA gene amplicon sequencing as complementary methods for the investigation of the cheese microbiota

**DOI:** 10.1186/s12866-022-02451-y

**Published:** 2022-02-07

**Authors:** Matthias Dreier, Marco Meola, Hélène Berthoud, Noam Shani, Daniel Wechsler, Pilar Junier

**Affiliations:** 1 Agroscope, Schwarzenburgstrasse 161, CH-3003 Bern, Switzerland; 2 Laboratory of Microbiology, University of Neuchâtel, Emile-Argand 11, CH-2000 Neuchâtel, Switzerland; 3 Department of Biomedicine, Applied Microbiology Research, University of Basel, Basel, Switzerland; 4 Clinical Bacteriology and Mycology, University Hospital Basel, Basel, Switzerland; 5 Swiss Institute for Bioinformatics, Basel, Switzerland

## Abstract

**Background:**

Next-generation sequencing (NGS) methods and especially 16S rRNA gene amplicon sequencing have become indispensable tools in microbial ecology. While they have opened up new possibilities for studying microbial communities, they also have one drawback, namely providing only relative abundances and thus compositional data. Quantitative PCR (qPCR) has been used for years for the quantification of bacteria. However, this method requires the development of specific primers and has a low throughput. The constraint of low throughput has recently been overcome by the development of high-throughput qPCR (HT-qPCR), which allows for the simultaneous detection of the most prevalent bacteria in moderately complex systems, such as cheese and other fermented dairy foods. In the present study, the performance of the two approaches, NGS and HT-qPCR, was compared by analyzing the same DNA samples from 21 Raclette du Valais protected designation of origin (PDO) cheeses. Based on the results obtained, the differences, accuracy, and usefulness of the two approaches were studied in detail.

**Results:**

The results obtained using NGS (non-targeted) and HT-qPCR (targeted) show considerable agreement in determining the microbial composition of the cheese DNA samples studied, albeit the fundamentally different nature of these two approaches. A few inconsistencies in species detection were observed, particularly for less abundant ones. The detailed comparison of the results for 15 bacterial species/groups measured by both methods revealed a considerable bias for certain bacterial species in the measurements of the amplicon sequencing approach. We identified as probable origin to this PCR bias due to primer mismatches, variations in the number of copies for the 16S rRNA gene, and bias introduced in the bioinformatics analysis.

**Conclusion:**

As the normalized microbial composition results of NGS and HT-qPCR agreed for most of the 21 cheese samples analyzed, both methods can be considered as complementary and reliable for studying the microbial composition of cheese. Their combined application proved to be very helpful in identifying potential biases and overcoming methodological limitations in the quantitative analysis of the cheese microbiota.

**Supplementary Information:**

The online version contains supplementary material available at 10.1186/s12866-022-02451-y.

## Introduction

Molecular biology methods are increasingly replacing classical microbiological methods for the investigation of microbial communities in food products. More specifically, new developments in sequencing technology have made the use of next-generation sequencing (NGS) more affordable and widely applicable. Especially, 16S rRNA gene amplicon sequencing is becoming increasingly widespread to investigate the composition of bacterial communities in a variety of ecosystems. Nevertheless, the optimization and validation of such approaches can be a challenging task, especially because many different aspects have to be considered to achieve reliable results [1, 2]. For instance, the comparison of commonly used sequencing platforms, databases, and classification algorithms applied to mock communities consisting of bacterial species found in dairy products has revealed significant variations in the observed bacterial community compositions [3]. Furthermore, the accurate taxonomic classification of species in complex samples remains a challenging task, which depends on many factors, such as the selected primers for the variable 16S rRNA gene region, the taxonomy assignment method, and the database used [4–7]. Ecosystem-based databases for taxonomy assignment can achieve higher resolution at the species level [8–11] as shown by an improvement in species level classification obtained with a specific and manually curated database for milk and cheese analysis as compared to more general databases [9].

Apart from the comparison of the sequencing platforms and the bioinformatics analysis, it is relatively complex to validate the results of NGS with other approaches, as most other methods do not provide comparable in-depth data. For instance, a study comparing traditional culture methods and NGS in fecal and hypopharyngeal samples of healthy children found that the second method identified 7 to 20 times more unique species [12]. Most frequently, the 16S rRNA gene amplicon and shotgun sequencing methods have been compared to each other [13–17]. However, a large study of microbial communities in lakes in Brazil has reported a weak correlation and major differences in taxonomic diversity and abundance data between the two methods, with amplicon sequencing detecting significantly more phylum- and family-level diversity [17].

An inherent limitation of the amplicon sequencing method is its compositional information in terms of the relative abundances of the individual members of the community (operational taxonomic units [OTUs], amplicon sequence variants [ASVs], and taxa). However, quantifying different members of complex microbial communities is crucial for differential abundance analysis, such as to better understand the temporal dynamics of microbial communities and, in the case of food microbiology, to identify taxonomic groups that impact quality by causing off-flavors in fermented foods when reaching certain levels [18, 19]. Quantitative real-time PCR (qPCR) is one of the most widely used methods to precisely quantify bacteria in complex ecosystems. A difficulty posed for the quantification of specific taxa in complex systems is that specific primer systems have to be designed, which can be a very labor-intensive task. In addition, the low throughput of conventional qPCR systems is a limitation that adds to high labor and material costs. The development of high-throughput qPCR (HT-qPCR) has led to a reduction in the work load and material costs (i.e., PCR chemicals) and has opened up new fields of application. These include the investigation of synthetic bacterial soil communities [20], the determination of functional genes in soils [21], the quantification of pathogens in spiked feces and environmental water samples [22], the investigation of microbial diversity in the intestines of piglets [23], and the quantification of bacteriophages of the species *Lactococcus* (*Lc.*) *lactis* and *Leuconostoc* spp. in cheese milk [24].

In food microbiology, qPCR and NGS have been increasingly used in recent years to better understand the microbial composition of various foods [25, 26]. Fermented foods are composed of an often limited number of core species selected by the strictly controlled conditions during the production process and the limited supply of nutrients, pH, and temperature. Therefore, they are particularly suitable for the study of the bacterial communities by qPCR. However, HT-qPCR has only recently been used for the systematic analysis of fermented foods [27], while numerous studies have applied NGS [26, 28–30].

The aim of this study was to compare the performance of a 16S rRNA gene amplicon sequencing approach to a recently developed HT-qPCR method for the analysis of cheese DNA samples. Raclette du Valais protected designation of origin (PDO) cheeses were selected for their higher microbial diversity compared to other cheeses, resulting from the use of raw milk and the low scalding temperature. To this end, we analyzed bacterial community composition in DNA samples from 21 Raclette du Valais PDO cheeses originating from the same number of different cheese producers distributed in the Canton of Valais (Switzerland) using both approaches and compared the results.

## Materials and Methods

### Sampling

In this study, 21 Raclette du Valais PDO cheese loaves (S01-S21) produced in the same number of different cheese dairies were collected after 120 days of ripening. Twenty of the cheeses were manufactured on the same date, and one cheese (S17) was manufactured 11 days later. Raclette du Valais PDO is a semi-hard, smear-ripened, full fat cheese produced from raw milk and mainly consumed in melted form after a ripening time of at least three months. For the production of Raclette du Valais PDO, the 21 cheese dairies followed the specifications of the Raclette du Valais AOP association [31]. With regard to the use of starters and additional cultures, a mesophilic starter culture of lactic acid bacteria is added consisting of strains of *Lc. lactis* subsp. *lactis*, *Lc. lactis* subsp. *cremoris,* and *Lc. lactis* subsp. *lactis* biovar *diacetylactis*; and, depending on the production site, the thermophilic lactic acid bacteria *Streptococcus thermophilus* and/or *Lactobacillus helveticus* are occasionally also added to the cheese milk [32].

### DNA extraction

Bacterial pellets from cheese were obtained by adding 10 g of cheese to 90 ml modified peptone water (10 g/l peptone from casein, 5 g/l sodium chloride, 20 g/l trisodium citrate dihydrate, pH 7.0) and incubating for 10 min at 40 °C. The sample was then homogenized for 3 min in a Stomacher (Masticator, IUL Instruments, Königswinter, Germany). A 50 μl volume of 10% (w/v) sodium dodecyl sulfate was then added to 10 ml of the homogenate, which was then thoroughly mixed and centrifuged (4000 × g, room temperature, 30 min). Cell lysis and genomic DNA extraction were performed using the EZ1 DNA Tissue kit and a BioRobot® EZ1 workstation (Qiagen, Hilden, Germany). Briefly, bacterial pellets were resuspended in 250 μl G2 buffer (EZ1 DNA Tissue kit), transferred in 0.5 ml skirted tubes containing 100 mg 0.1 mm low binding zirconium beads (OPS Diagnostics, Lebanon, NJ, USA), and shaken 15 s at medium speed in a bead ruptor (Omni International Inc., Kennesaw, GA, USA). Cell lysates were then processed by the BioRobot® EZ1 workstation. Genomic DNA was eluted in a volume of 100 μl, and the concentration was measured using a NanoDrop® ND-1000 spectrophotometer (NanoDrop Technologies, Thermo Fisher Scientific, Waltham, MA, USA).

### HT-qPCR primers

The primers used for HT-qPCR in this study were described in a previous study [27]. Briefly, 24 target species/subspecies were selected based on a review of the literature and our own preliminary results from the 16S rRNA gene amplicon sequencing of Gruyere and the Raclette du Valais PDO cheeses considered in this study (unpublished data). The selection criteria for the target species were the relative abundance and frequency of detection as well as known impacts on cheese quality.

### HT-qPCR standards

The standards for quantification in the HT-qPCR system were produced using standard calibration curves of gBlock™ Gene Fragments (Integrated DNA Technologies, LubioScience, Switzerland), described in detail previously [27]. Copy numbers for quantification were calculated using standard calibration curves ranging from 10^7^ to 10^3^ copies/μl.

### Microfluidic HT-qPCR

HT-qPCR was performed using a 192.24 Dynamic Array integrated fluidic circuit (IFC; Fluidigm Corporation, San Francisco, CA, USA). The assay mix consisted of 3 μl 2× Assay Loading Reagent (Fluidigm Corp.) added to 3 μl primer mix (forward and reverse, 10 μM). A sample pre-mix was prepared by combining 3 μl 2× SsoFast™ EvaGreen® Supermix with low ROX (Biorad, Cressier, Switzerland) and 0.3 μl 192.24 Delta Gene Sample Reagent (Fluidigm Corp.). Finally, 2.7 μl of each sample were added to 3.3 μl sample pre-mix. The IFC was loaded according to the manufacturer’s instructions [33]. Briefly, 3 μl of each assay and 3 μl of each sample were distributed to the respective inlet, and the IFC was loaded using the Juno Load Mix 192.24 GE script. The loaded IFC was transferred to the Biomark instrument and run with the GE 192x24 PCR + Melt v2 program, as follows: hot start 95 °C for 1 min, followed by 30 cycles of denaturation at 96 °C for 5 s, and annealing and elongation at 60 °C for 20 s. A melting curve analysis was performed with a temperature increase of 1 °C per 3 s from 60 to 95 °C.

### HT-qPCR data analysis

The results from the 192.24 Dynamic Array IFCs were analyzed with the Fluidigm Real-Time PCR Analysis Software version 4.5.2 (Fluidigm Corp.) as described in a previous study [27]. The melting curve peak threshold was set to 0.025 -dRn/dT based on a visual inspection of the baseline fluorescence. All reactions flagged by the Real-Time PCR Analysis Software were interpreted as negative results. The copies/μl of the specific targets were calculated for each reaction using the standard calibration curves, and all reactions below an 800 copies/μl cut-off were interpreted as negative, as recommended by the manufacturer [34]. Average copies/μl were only calculated if at least two of three reactions were positive; otherwise, the results were interpreted as negative.

### 16S rRNA gene amplicon sequencing

Amplicon libraries were prepared using the unidirectional fusion method (Thermo Fisher Scientific, Waltham, MA, USA). PCR of the V1–V2 16S rRNA gene region was performed in 50 μl reactions using 4 μl of DNA, 0.1 μM primer NGS_ABCxF27 (5′- CCA TCT CAT CCC TGC GTG TCT CCG ACT CAG |Barcode X| AG AGT TTG ATC MTG GCT CAG − 3′) and 0.1 mM primer NGS_trP1_355 (5′- CCT CTC TAT GGG CAG TCG GTG ATG CWG CCT CCC GTA GGA GT − 3′), and 45 μl Platinum™ PCR SuperMix High Fidelity (Thermo Fisher Scientific, Waltham, MA, USA). The amplification was carried out as follows: 94 °C for 2 min, followed by 18 cycles of 94 °C for 30 s, 55 °C for 30 s, and 68 °C for 30 s. All amplicons were purified using AMPure XP beads (Beckman Coulter, Brea, CA, USA) with a bead-to-DNA ratio of 1.8. The quality control and quantification of the amplicon library was performed using an Agilent 2100 Bioanalyzer (Agilent Technologies, Santa Clara, CA) and the High Sensitivity DNA Assay. Afterwards, all amplicons were pre-diluted and equimolarly pooled to a 40 pM final library. Template preparation, chip loading, and sequencing were performed according to the manufacturer’s instructions using Ion Chef™ System and Ion S5™ System and an Ion530 Chip (Thermo Fisher Scientific, Waltham, MA, USA).

### 16S rRNA gene amplicon sequencing data analysis

The raw sequences, with an average length of 320 bp, were primer trimmed and quality filtered (maxEE = 15, truncQ = 6, maxN = 0, n = 1e+06, minLen = 100, maxLen = 460) in DADA2 [35]. Amplicon sequence variances (ASVs) were obtained in DADA2 with the parameter POOL = “pseudo.” Taxonomic annotation was performed using DAIRYdb v1.2.4 [9] with IDTAXA [36]. Biostatistical analyses were done using the PHYLOSEQ package [37] in R v4.0.2 [38]. Copy number normalization was based on the copy number information available in the Ribosomal RNA Database (rrnDB, version 5.7, January 18, 2021, [39]).

### Method comparison data analysis

For the data analysis, we used the following Python packages: Jupyter-notebook v6.2.0 [40] with Python v3.9.2 and IPython v7.21.0 [41], NumPy v1.20.1 [42], seaborn v0.11.1 [43], pandas v1.2.3 [44], SciPy v1.6.1 [45], Matplotlib v3.3.2 [46], statsmodels v0.12.2 [47], and rpy2 v3.4.3. Further, R v4.0.3 [38] and the metacal v0.2.0 package [48] were used for bias estimation (see below). The data analysis was performed as outlined in the htqpcr_ngs_comparison_R.ipynb notebook available in Additional file 1 and the Github repository [49].

Briefly, the taxonomic assignments and number of reads from the 16S rRNA gene amplicon sequencing (NGS) analysis data for the 21 Raclette du Valais PDO samples were extracted from the data set. The most prevalent species in the NGS results were defined as species detected in more than 30% of the cheese samples. Relative species abundance was calculated for each cheese DNA sample considering all members of the community to create plots representing the community composition. HT-qPCR analysis was performed using the HTqPCR_dataparser.py script. Further, the data for the two *Lc. lactis* subspecies from HT-qPCR were grouped to *Lc. lactis*. For the bias estimation, the data from both methods were filtered to consider only the 15 bacterial species/groups measured by both methods (shared positive). The NGS data were defined as the observed category, and only read counts for species also detected by the HT-qPCR approach (reference) were included. Further, pseudocounts (=1) were added in the observed data (NGS) for species only detected in the reference data (HT-qPCR). Bias estimates were calculated using metacal with the number of reads and copies of the 15 investigated bacterial species/groups measured by both methods as input. A corrected data set was made by grouping the data for *Lactiplantibacillus pentosus* and *Lactiplantibacillus plantarum* for NGS and for *L. plantarum* and *Lactiplantibacillus paraplantarum* for the HT-qPCR to a common *L. plantarum* group category.

### Bias estimation

The model for bias estimation is described in detail in [48]. Briefly, the assumption of this bias model is that the bias is caused by the different efficiencies for the given measurement (relative or absolute abundance) of different species. The bias estimates are calculated from taxon proportions to make the bias independent of the sample’s composition. The systematic difference between measurements from different methods can be estimated by the difference in their biases. If the actual composition is not known, but a reference composition is considered as the true composition, this differential bias is equivalent to the bias of the method under investigation. A point estimate of the bias (the ratio of the efficiency of a species to the geometric mean efficiency of all species) for each species with known (reference) abundance can then be calculated for the samples. Geometric standard errors were estimated from 1000 bootstrap replicates.

### Reference sequence alignments

Representative genomes of the reference species were downloaded from the National Center for Biotechnology Information (NCBI). The 16S rRNA gene sequences were extracted using Barrnap V 0.9 [50]. Non-redundant sequences of the V1–V2 region of the 16S rRNA gene were aligned using PRANK V .150803 [51].

### Construction of phylogenetic tree from reference sequences and ASVs

The reference 16S rRNA gene sequences for the *L. plantarum* group species were extracted from the DAIRYdb v1.2.4 fasta file [52]. ASVs assigned to the *L. plantarum* group species were filtered. The sequences of the V1–V2 region of the 16S rRNA gene were aligned using PRANK V .150803, and the resulting multiple sequence alignment was subjected to a rapid bootstrap analysis using RAxML [53]. The best-scoring maximum likelihood tree was visualized using iTOL [54].

## Results and Discussion

### HT-qPCR and 16S rRNA gene amplicon sequencing results

**Fig. 1:**
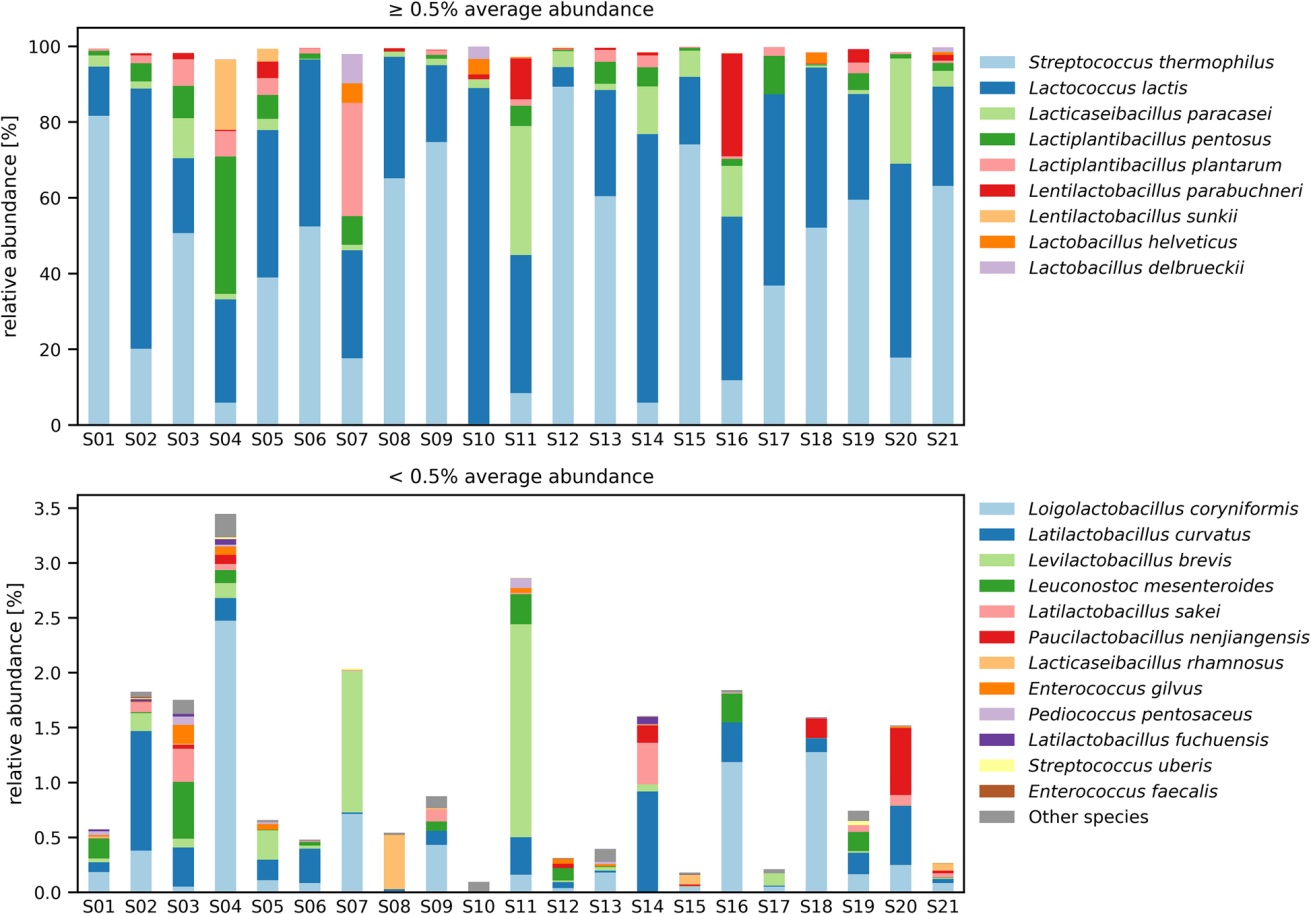
Bacterial community composition determined by 16S rRNA gene amplicon sequencing. Species detected in Raclette du Valais PDO cheese DNA samples (S01-S21) with an average relative abundance above 0.5% are shown in the upper panel and the species with a lower average abundance in the lower panel. The relative abundance of the 21 species detected in more than 30% of the samples are depicted with the species name; the other 26 taxa were classified as other species

The average sequencing depth for 16S rRNA gene amplicon sequencing was 471,184 reads (range: 361496–632,269). In total, 9,894,860 reads were classified to 233 ASVs. These ASVs were assigned to 47 different sequence groups, and 45 of these were classified to the species level, while for two ASV groups only a classification to the family level (*Ruminococcaceae, Streptococcaceae*) was possible. Four core species were detected in all 21 cheese samples. These species were *Lacticaseibacillus paracasei*, *Lc*. *lactis*, *L. helveticus*, and *S*. *thermophilus*. Ten species were present in more than 80% of the samples and belonged to either the *Lactobacillaceae* or the *Streptococcaceae* families. The 21 most prevalent species (occurring in at least 30% of cheeses) represented on average 99.96% (range 99.79–100%) of all the reads and included species from the *Lactobacillaceae*, *Leuconostocaceae*, *Enterococcaceae,* and *Streptococcaceae* families (Fig. 1).

**Fig. 2:**
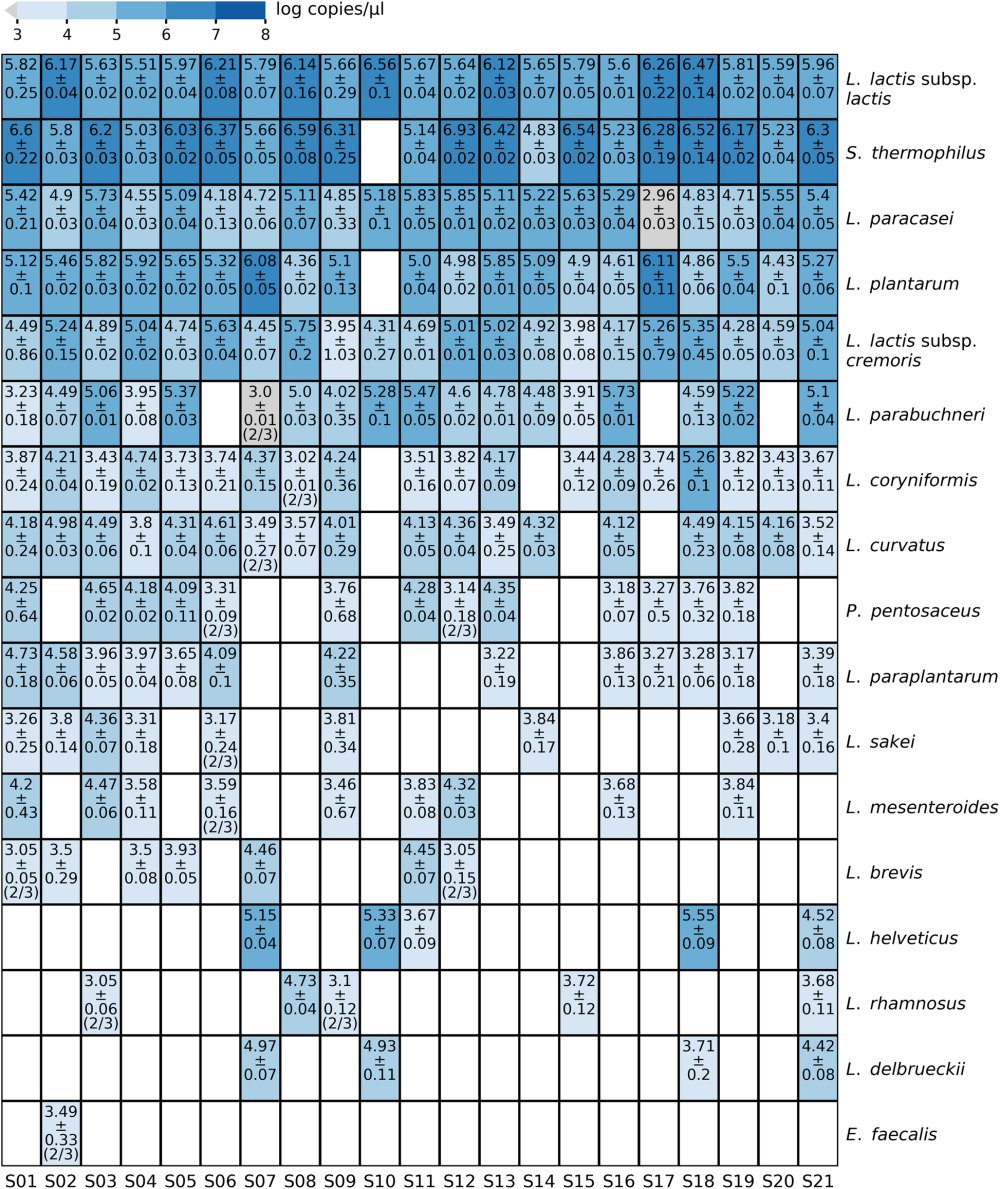
Heatmap of HT-qPCR results. The heatmap annotation depicts the average log copies/μl and the standard deviation of technical triplicates. When not all samples were positive, the number of positive samples out of the total number of samples is given in brackets

The HT-qPCR system consisted of specific primer pairs targeting 24 different bacterial species/subspecies, of which a total of 17 species/subspecies were quantified in at least one of the cheese DNA samples (Fig. 2). *Lc*. *lactis* subsp. *lactis*, *Lc*. *lactis* subsp. *cremoris,* and *L*. *paracasei* were detected in all samples, while *S. thermophilus* and *L. plantarum* were detected in all but one sample (sample S10). *Lc*. *lactis* subsp. *lactis* was the dominant subspecies in all samples. Surprisingly, *L. helveticus* was detected only in five samples by HT-qPCR. Three additional species (*Lentilactobacillus parabuchneri*, *Loigolactobacillus coryniformis,* and *Latilactobacillus curvatus*) were detected in more than 80% (17) of the samples, while *L. paraplantarum* was detected in 62% (13) of the cheese samples. The seven species in the HT-qPCR system that were not detected in any of the cheeses examined corresponded to *Clostridium tyrobutyricum*, *Enterococcus durans*, *Enterococcus faecium*, *Lacticaseibacillus casei*, *Limosilactobacillus fermentum*, *Pediococcus acidilactici*, and *Propionibacterium freudenreichii*.

The sampled Raclette du Valais PDO originated from commercial batches of good-quality cheeses and thus did not exhibit any sensory-perceptible quality defects. Therefore, the absence or at least very low relative abundance of bacterial species responsible for cheese quality defects, such as *C. tyrobutyricum* (butyric acid fermentation) or *E. durans* and *E. faecium* (potential tyramine producers), was expected. Moreover, in the production of Raclette du Valais PDO, no adjunct cultures containing *L*. *casei*, *L*. *fermentum*, *P. acidilactici,* or *Propionibacterium freudenreichii* are used, therefore the detection of these species depends on whether they were present in the raw milk.

To our knowledge, no study has investigated Raclette du Valais PDO cheese using 16S rRNA amplicon sequencing and HT-qPCR yet. Other semi-hard cheese varieties made from raw milk have been investigated by 16S rRNA gene amplicon sequencing, but only few studies report the community compositions at a species level resolution. In two Raclette-type cheeses made from raw milk, analyzed with the same NGS approach as in the present study, the dominant species were *Lc. lactis*, *L. plantarum/pentosus* and *Weissella paramesenteroides*, with a relative abundance of *Lc. lactis* exceeding 50% [29]. In the present study *W. paramesenteroides* was also detected in five samples with a low abundance (<0.085%). A recent study identified *Lactobacillus delbrueckii*, *Lacticaseibacillus rhamnosus*, *L*. *casei*, *L*. *helveticus* and *L. fermentum* as the most abundant and prevalent species in Grana Padano cheeses [55]. In contrast to Raclette du Valais the scalding temperature is higher (56 °C) and the natural whey starters are dominated by *S. thermophilus*, *L. delbrueckii* and *L. helveticus* and lower proportions of *L. fermentum* [56]. Ten species, namely, *S. thermophilus, Lc. lactis, L. rhamnosus, Latilactobacillus sakei, L. coryniformis, Pediococcus pentosaceus, W. paramesenteroides*, *L. plantarum, L. (para-)casei* and *Weissella hellenica* (sample S20 only) with an average relative abundance above 0.5% in three samples of Danish cheeses made from raw milk ripened for 56 days [57], were also found in our study of Raclette du Valais. In contrast to Grana Padano, the scalding temperature used for the manufacture of this cheese was 39 °C, which is more comparable to the temperature used for Raclette du Valais manufacture (36 °C). *Ligilactobacillus acidipiscis* and *Staphylococcus saprophyticus* were taxa of the indigenous microbiota exclusively found in the Danish cheeses. These results support the observations of many studies showing that parameters such as the type and origin of milk, milk treatment, and the type of ripening significantly influence the microbiota in ripened cheese (reviewed in [58, 59]).

### Comparison of HT-qPCR and 16S rRNA gene amplicon sequencing results

**Fig. 3:**
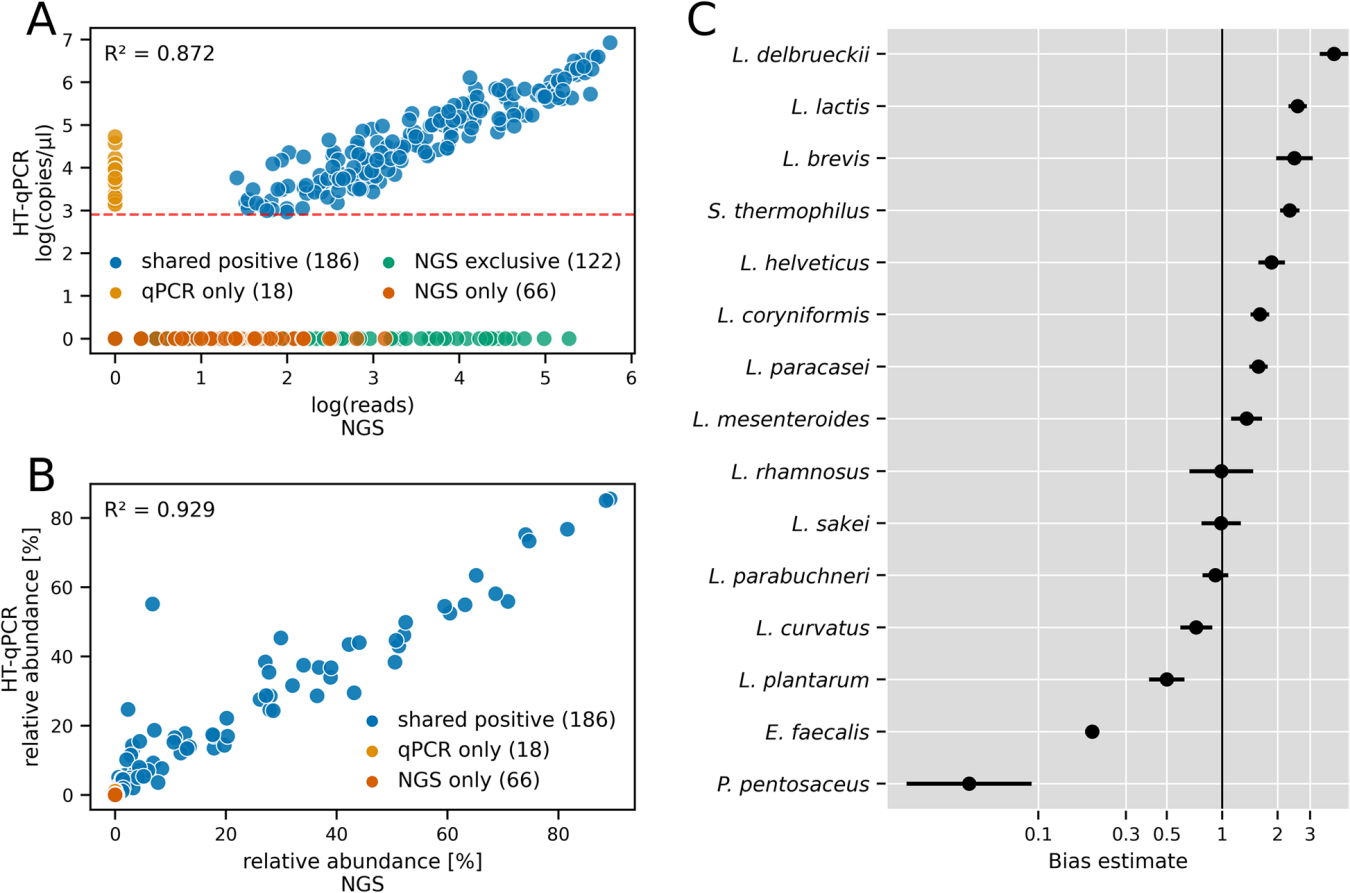
Comparison of HT-qPCR and 16S rRNA gene amplicon sequencing (NGS). **A** Logarithmic HT-qPCR data (y-axis) and logarithmic count data corresponding to the NGS approach (x-axis). The red line depicts the threshold of 800 copies/μl used for the HT-qPCR data analysis. Shared positive: Measurements obtained with both methods. qPCR only/NGS only: Measurements obtained solely by one of the two methods, HT-qPCR or NGS, respectively. NGS exclusive: Measurements of taxa by NGS for which no HT-qPCR assay was available. The number of observations for each group is given in brackets. **B** Direct comparison of the relative abundance data of HT-qPCR (copies/μl) on the y-axis and NGS (reads) on the x-axis. The taxa that were exclusively detected by NGS (NGS exclusive) were not considered. **C** Plot of the bias point estimates ± two geometric standard errors calculated for the NGS approach using the HT-qPCR approach as a reference method. Only the data of the shared positive measurements were used for the bias estimation

The HT-qPCR analysis results represent, after comparison with a standard curve, the number of copies of the species-specific single copy gene per μl of sample, while the amplicon sequencing results correspond to the relative abundance of taxa based on the number of reads with respect to the total number of reads of the corresponding V1–V2 16S rRNA gene region. The fundamental differences in the resulting data (absolute or relative abundances) and the data analysis (standard or compositional) make a direct comparison between the two methods challenging. The first attempt to compare the performance of the two methods qualitatively was based on a comparison of the measured copy numbers versus the number of NGS reads (Fig. 3A). Given that the HT-qPCR system contained specific primer pairs that were able to discriminate between the subspecies of *Lc. lactis* (subsp. *lactis* and subsp. *cremoris*), while the amplicon sequencing was not able to discriminate these subspecies, the data for the two subspecies were pooled to account for the total number of *Lc. lactis* for the comparison of the methods. The data points in Fig. 3A were divided into four groups. The group “shared positive” represents measurements for the 15 species that were covered and detected by both methods. The second and third groups (“qPCR only” and “NGS only”) included measurements in which the same 15 species were detected either by HT-qPCR or NGS. The measurements for all other taxa that were not covered by the selected HT-qPCR assays were classified as “NGS exclusive.” The detection of a larger number of exclusive taxa using the 16S rRNA gene amplicon sequencing method was expected given the non-targeted nature of the NGS approach. However, in the case of the two species *L. paraplantarum* and *P. pentosaceus* that were detected solely by HT-qPCR, the unexpected outcome may indicate errors or bias in the analysis of the NGS results. For the log-transformed data (Fig. 3A) and the relative abundance data (Fig. 3B) of the “shared-positive” group, positive linear correlations (R^2^ = 0.872 and R^2^ = 0.929, respectively) were observed. The relative abundance data in Fig. 3B indicate that the qualitative disagreement between the methods was mainly due to species with low relative abundance, which were detected only by the NGS method.

A summary of the frequencies of the detection of the most prevalent species by both methods is shown in Table 1. In 66 cases, NGS exclusively detected species also targeted by HT-qPCR (“NGS only”), and the relative abundances for these species were in a range between 0.0002 and 0.322%. For example, *L. helveticus* was detected in all samples by NGS, while HT-qPCR did not detect *L. helveticus* in 16 samples with low relative abundances (0.004–0.043%). Similarly, *L. delbrueckii* was exclusively detected by NGS in 15 cheese DNA samples with relative abundances between 0.002–0.025%. In contrast, *L. paraplantarum* and *P. pentosaceus* were detected in a higher number of samples by HT-qPCR than by NGS. *L. paraplantarum* was detected in 13 samples by HT-qPCR with abundances in a range between 1472 and 53,306 copies/μl, whereas this species was never detected by NGS in any of the analyzed samples. In five samples, *P. pentosaceus* was exclusively detected by HT-qPCR with a range of 1389–6542 copies/μl. Reciprocally, *P. pentosaceus* was exclusively detected by NGS in one sample (relative abundance 0.0055%).

**Table 1:** Detected species and average relative abundance for the HT-qPCR and 16S rRNA gene amplicon sequencing (NGS) in 21 Raclette du Valais PDO cheese samples

	qPCR			NGS		
	Count	Avg. abund. [%]	SD	Count	Avg. abund. [%]	SD
***Lacticaseibacillus paracasei***	21	8.26	10.59	21	6.34	9.10
***Lactococcus lactis***	21	32.79	18.17	21	37.18	20.40
***Streptococcus thermophilus***	20	42.03	25.51	21	42.24	28.20
***Lactiplantibacillus plantarum***	20	12.00	14.76	20	3.47	6.56
***Loigolactobacillus coryniformis***	19	0.60	0.95	19	0.42	0.62
***Lentilactobacillus parabuchneri***	18	4.96	9.28	20	2.68	6.27
***Latilactobacillus curvatus***	18	0.77	0.85	19	0.26	0.30
***Lactobacillus helveticus***	5	3.28	2.48	21	0.63	1.47
***Leuconostoc mesenteroides***	9	0.31	0.22	14	0.13	0.15
***Lactobacillus delbrueckii***	4	1.58	1.54	19	0.66	1.89
***Levilactobacillus brevis***	7	0.47	0.61	16	0.26	0.55
***Latilactobacillus sakei***	10	0.24	0.25	12	0.10	0.12
***Pediococcus pentosaceus***	13	0.41	0.43	9	0.03	0.03
*Lactiplantibacillus pentosus*	0			20	5.21	7.91
***Lacticaseibacillus rhamnosus***	5	0.24	0.36	13	0.05	0.14
*Enterococcus gilvus*	0			15	0.03	0.05
*Lactiplantibacillus paraplantarum*	13	0.38	0.42	0		
*Lentilactobacillus sunkii*	0			10	2.23	5.85
*Streptococcus uberis*	0			10	0.01	0.01
*Latilactobacillus fuchuensis*	0			9	0.02	0.02
***Enterococcus faecalis***	1	0.11		7	0.00	0.00
*Paucilactobacillus nenjiangensis*	0			8	0.14	0.20
Other species	0			20	0.05	0.06

The relative abundance of the 21 species detected in more than 30% of the samples by NGS and *Lactiplantibacillus paraplantarum* exclusively detected by HT-qPCR are depicted with the species name; the other 26 taxa were classified as other species

The relatively high detection limit of 800 copies/μl is most likely responsible for the inability of the HT-qPCR method to detect low abundance species. The number of target gene copies presumably also has an influence on sensitivity. The HT-qPCR system targets specific single-copy genes, while according to the rrnDB database, most of the prevalent species contain about five copies (range: 3–9) of the 16S rRNA gene (Additional file 2). The sensitivity of the HT-qPCR assays is further limited by the nanoliter-scale reactions used in the microfluidic qPCR system compared to standard qPCR methods. Apart from these differences for low abundance species, the 15 species included in the method comparison represented 93.84% (range: 44.69–99.93%) of the reads from the NGS analysis (Table 2), indicating that the most dominant members of the microbial population in the 21 Raclette du Valais PDO samples was covered by both approaches. Samples for which the coverage was below the average (S03, S04, S05, S07, and S17) all showed above-average relative abundances of *L. pentosus*. In addition, sample S04 also showed an above-average relative abundance of *Lentilactobacillus sunkii*. Besides these two species, the other species not targeted by the HT-qPCR system accounted for only 0.13% (range: 0.01–0.63%) of the NGS reads. Neither *L. pentosus* nor *L. sunkii* were included in the HT-qPCR system since no validated primers for these species were available. For future studies, it would be beneficial to design specific primers for these species to enable the quantification of these common species in cheese using the HT-qPCR approach. *L. pentosus* has already been isolated from milk and cheese [58, 60]. *L. sunkii* was originally isolated from sunki, an unsalted Japanese fermented food, and has already been detected in kefir biofilms [61, 62]. However, to our knowledge, the first detection of *L. sunkii* in cheese was reported only recently in an NGS study of Grana Padano cheese [55].

**Table 2:** Relative abundance data of species detected by 16S rRNA gene amplicon sequencing

	Shared species		Other species		***L. pentosus***	***L. sunkii***
	[%]	[n]	[%]	[n]	[%]	[%]
S01	98.73	13	0.03	3	1.24	0.01
S02	95.06	12	0.09	6	4.83	0.02
S03	91.16	14	0.36	6	8.47	
S04	44.69	14	0.44	11	36.27	18.6
S05	90.24	13	0.07	4	6.33	3.36
S06	98.62	12	0.01	5	1.37	
S07	92.47	12	0.01	1	7.52	
S08	99.93	10	0.02	3	0.05	
S09	98.89	12	0.11	3	1.00	
S10	99.91	7	0.09	1		
S11	94.59	15	0.05	3	5.36	
S12	99.31	13	0.09	5	0.33	0.27
S13	94.06	12	0.14	4	5.81	
S14	94.76	13	0.23	5	5.01	
S15	99.26	10	0.03	5	0.71	
S16	98.11	11	0.02	4	1.86	
S17	89.88	11	0.04	2	10.08	
S18	99.5	11	0.18	3	0.31	
S19	95.44	14	0.13	6	4.43	
S20	98.24	10	0.63	5	1.12	0.01
S21	97.85	13	0.03	7	2.11	

Percentage of reads assigned to species also covered by HT-qPCR (shared) and other species that were not covered by HT-qPCR. The two species, *Lactiplantibacillus pentosus* and *Lentilactobacillus sunkii,* with the largest overall abundance are shown separately

### Bias estimation

Over the 21 investigated cheese samples, the two methods show a high degree of qualitative agreement (detected or undetected) and a strong correlation between the measurements for the species covered by both approaches (Fig. 3 A and B). However, the correlation of relative abundance data for the same samples measured by two different methods is not a suitable indicator for the agreement or disagreement of the methods, since a high correlation can be expected for two methods measuring similar properties in the same samples. To examine the differences between the two methods in more detail, point estimates of the bias for the 15 shared species were calculated for the NGS approach using the HT-qPCR approach as a reference method (Fig. 3C). A bias estimate value above 1 indicates an increased efficiency for NGS, while values below 1 indicate a decreased efficiency for the measurement of the species compared to the reference method (HT-qPCR). A strong positive bias was observed for *L. delbrueckii, Lc. lactis, Levilactobacillus brevis*, and *S. thermophilus*, while a strong negative bias was observed for *L. plantarum*, *Enterococcus faecalis,* and *P. pentosaceus*.

### Identification of possible causes for negative bias

Potential biases can be introduced at every step of the NGS protocols, from nucleic acid extraction, library preparation, and sequencing, to the bioinformatics analysis [1, 48]. McLaren et al. [48] have shown, using mock communities analyzed by NGS, that the largest influence on the total bias originates from DNA extraction, followed by PCR. In our study, we used the same DNA samples for the measurements by both methods, therefore bias due to DNA extraction did not contribute to the observed total bias. We performed a bioinformatics analysis to identify possible causes of the decreased efficiency of NGS for *L. plantarum* and *P. pentosaceus*. The analyses were not repeated for *E. faecalis* as the bias estimate was based on just a single measurement. Alignments of the V1–V2 region of the 16S rRNA gene sequences from the representative genomes have shown one or two nucleotides difference between *L. plantarum* and *L. pentosus* and only three or four nucleotides difference for *L. paraplantarum* (Supplementary Fig. S1 A, Additional file 3). Multiple copies of the 16S rRNA gene in the representative genomes of *L. plantarum* and *L. paraplantarum* were not identical and contained single nucleotide polymorphisms (SNPs). The DAIRYdb (v.1.2.4), used by IDTAXA for taxa assignment, contained four 16S rRNA gene reference sequences spanning the entire length of the V1–V2 region, two identical sequences for *L. pentosus* and one each for *L. plantarum* and *L. paraplantarum* (Supplementary Fig. S1 B, Additional file 3). The references for *L. plantarum* and *L. pentosus* contained only a single nucleotide transition. In the phylogenetic tree (Supplementary Fig. S1 C, Additional file 3) of the ASVs, two distinct clades could be identified, one for *L. paraplantarum* (purple) and one for *L. plantarum* and *L. pentosus* (green), including the genomic reference sequences and type strain sequences from the DAIRYdb. However, considering the high similarity of the reference sequences and the intra-strain SNPs in the representative genomes, it is likely that the assignment to *L. pentosus* or *L. plantarum* was based on single nucleotide differences. Nevertheless, it remains unclear why *L. paraplantarum* was never assigned by DAIRYdb-IDTAXA in the analysis pipeline.

These findings indicate the inability to differentiate *L. pentosus*, *L. plantarum,* and *L. paraplantarum* based on the selected primers for the V1–V2 variable region of the 16S rRNA gene as a source of underestimation bias using 16S rRNA gene sequencing. The difficulty in differentiating the species of the *L. plantarum* group has already been identified earlier [63–65]. Torriani et al. [66] reported that the partial sequences of the *recA*, *dnaK*, *tuf*, *hsp*60, and *pheS* genes allow a better differentiation of *L. plantarum, L. pentosus,* and *L. paraplantarum*.

Further investigations were undertaken to clarify whether other regions of the 16S rRNA gene would be better suited for the differentiation of these species. Primers targeting the V3–V4 variable regions of the 16S rRNA gene, which were used in recent studies of microbial populations in milk and cheese [30, 67], are even more problematic as this region displays 100% nucleotide identity for *L. plantarum, L. pentosus,* and *L. paraplantarum* in the representative genomes (Supplementary Fig. S2, Additional file 3). To prevent biased microbiota data, studies in fermented foods that rely solely on species identification based on 16S rRNA gene sequences should mention this limitation if *L. plantarum* group species are included or, alternatively, try to differentiate these species by additional analysis, such as multiplex PCR.

Regarding the investigation of the strong bias for *P. pentosaceus*, the potential reasons for PCR bias were examined. An alignment of the primer regions showed that only the sequence of *P. pentosaceus* had a potential mismatch at position 12 of the NGS_ABCxF27 primer (Supplementary Fig. S3, Additional file 3). The wobble base (M) at position 12 of the primer represents an adenine or cytosine, while the *P. pentosaceus* sequence at this position contains a thymine. Since it is a single mismatch and is not located at the 3′ end, it certainly does not prevent amplification but most likely can reduce primer efficiency. Notably, in one sample (S14), *P. pentosaceus* was detected by NGS but not by HT-qPCR. The relative abundance of *P. pentosaceus* in this sample was very low (0.0055%), and the total copies/μl of cheese sample S14 was low compared to the other samples. Looking at the qPCR raw data, we observed weak fluorescent signals in some reactions (technical triplicates) containing the *P. pentosaceus* assay; however, it was below the 800 copies/μl cut-off value used to improve the signal-to-background noise ratio.

Possible factors for positive bias were not investigated in detail here, but presumably the copy number of the 16S rRNA gene and PCR bias have an influence on the positive total bias. In addition, it must also be considered that even though we have chosen HT-qPCR as a reference for this comparison, the method is not independent of its own inherent bias.

### Bias correction

Since the 16S rRNA gene sequencing analysis work-flow was not able to differentiate between the three species of the *L. plantarum* group, those were combined into an *L. plantarum* group as a post-analysis bioinformatics correction. After this correction, the 15 investigated species (now including *L. pentosus*) accounted for 98.81% (range: 80.96–99.99%) of the reads of the NGS analysis. Only samples S04 and S05 had a coverage below the average, due to a high relative abundance (18.6 and 3.4%, respectively) of *L. sunkii* (Additional file 4). Another approach that has been investigated for partial correction of PCR bias is gene copy number normalization (GCN) of the 16S rRNA gene.

**Fig. 4:**
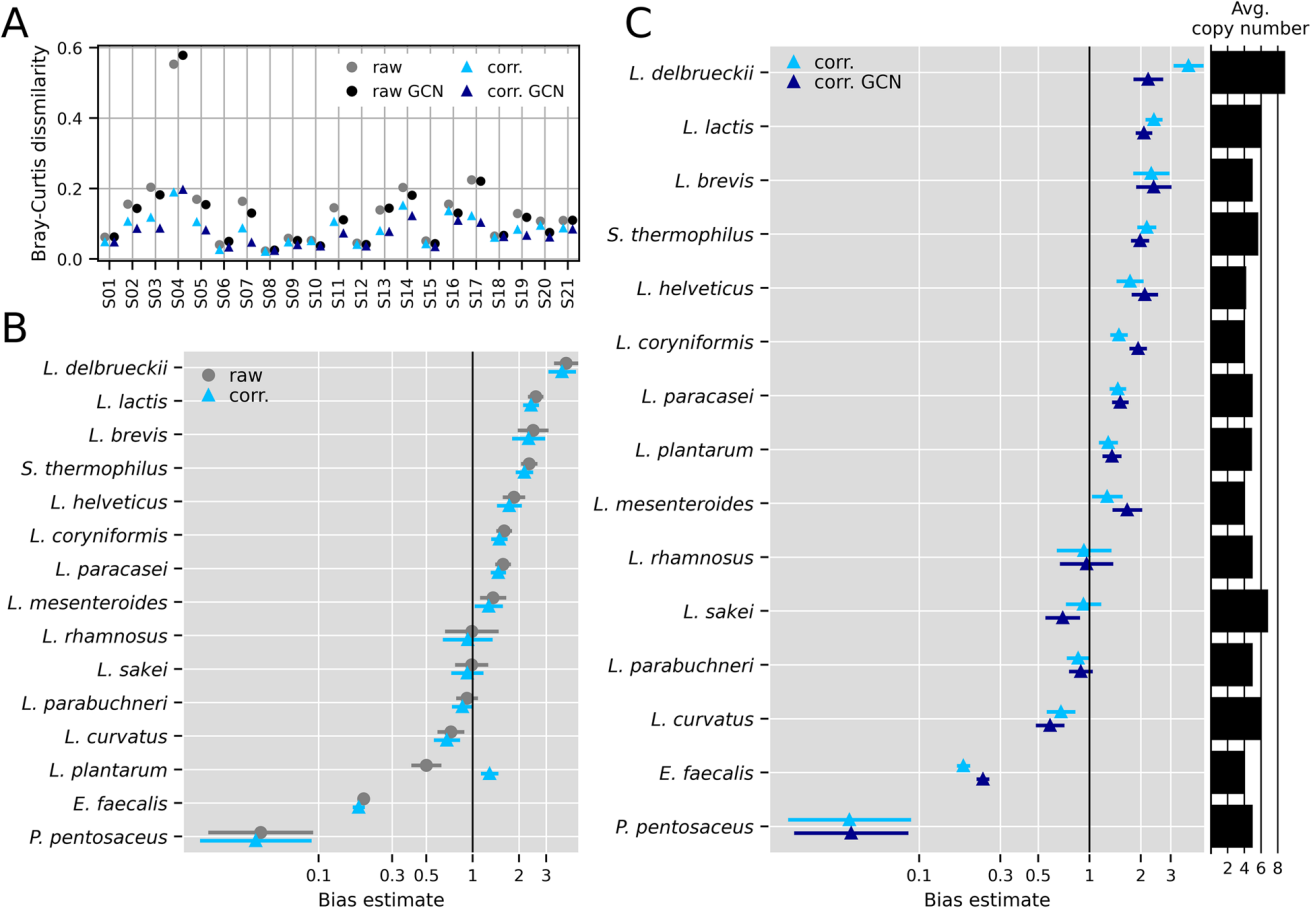
Bias estimates for raw and corrected data sets. **A** Bray-Curtis dissimilarity of relative abundance data of 16S rRNA gene amplicon sequencing (NGS) to HT-qPCR relative abundance data (reference). Depicted are the dissimilarities for the raw and the corrected *Lactiplantibacillus plantarum* group data sets without and with gene copy number normalization (GCN) for the 16S rRNA gene. **B** Bias point estimates ± two geometric standard errors before and after the correction of the assignments for the *L. plantarum* group species. **C** Bias point estimates ± two geometric standard errors after the correction of the assignments for the *L. plantarum* group species, with (corr. GCN) and without (corr.) 16S rRNA gene copy number correction

After the *L. plantarum* group correction, the Bray-Curtis dissimilarity relative to the HT-qPCR approach was decreased for all samples except S08 and S10 (Fig. 4A). Samples S08 and S10 were the only samples with a relative abundance of *L. pentosus* below 0.05%. Further, the Bray-Curtis dissimilarity was lower for the 16S copy number correction (black circles) compared to the raw data (gray circles) in most samples; only in samples S04, S06, S08, and S18 was the Bray-Curtis dissimilarity higher. A lower Bray-Curtis dissimilarity was calculated for the data with combined corrections for the *L. plantarum* group species and GCN (dark blue triangles) compared to the raw data. The bias for *L. plantarum* was noticeably improved for the *L. plantarum* group correction, while the bias for the other species barely changed (Fig. 4B). The bias changed from a decreased efficiency for the measurement of *L. plantarum* to an increased efficiency for the *L. plantarum* group species. This may be partially explained by the proportion of *L. pentosus* since *L. pentosus* was not measured by HT-qPCR.

For the GCN, the number of reads measured by NGS was divided by the average number of copies for each species. This approach had only a minor effect on the estimated biases (Fig. 4C). The average 16S rRNA copy number for the 15 investigated species was 5.3 copies; therefore, for species with higher copy numbers, the efficiency of the measurement decreased (*S. thermophilus, Lc. lactis, L. curvatus, L. sakei, L. delbrueckii*), while for species with lower copy numbers, the efficiency increased. The 16S rRNA GCN had only a major influence on the bias for species with a high average number of copies, namely *L. delbrueckii* (avg. copies: 8.9). McLaren et al. [48] have previously reported that the total bias was poorly explained by copy number correction for the mock communities used in their study. Improving the predictions for the composition of microbial communities based on 16S rRNA GCN, apart from mock communities, is still an unsolved problem [68]. Difficulties include, for example, that predictions of 16S rRNA copy numbers can be inaccurate and strongly differ between prediction tools for taxa with unknown numbers of copies of the 16S rRNA gene [69]. Other unresolved issues include varying copy numbers within the same genus or the intra-genomic heterogeneity of the 16S rRNA gene [70, 71].

### Potential and limitations

**Fig. 5:**
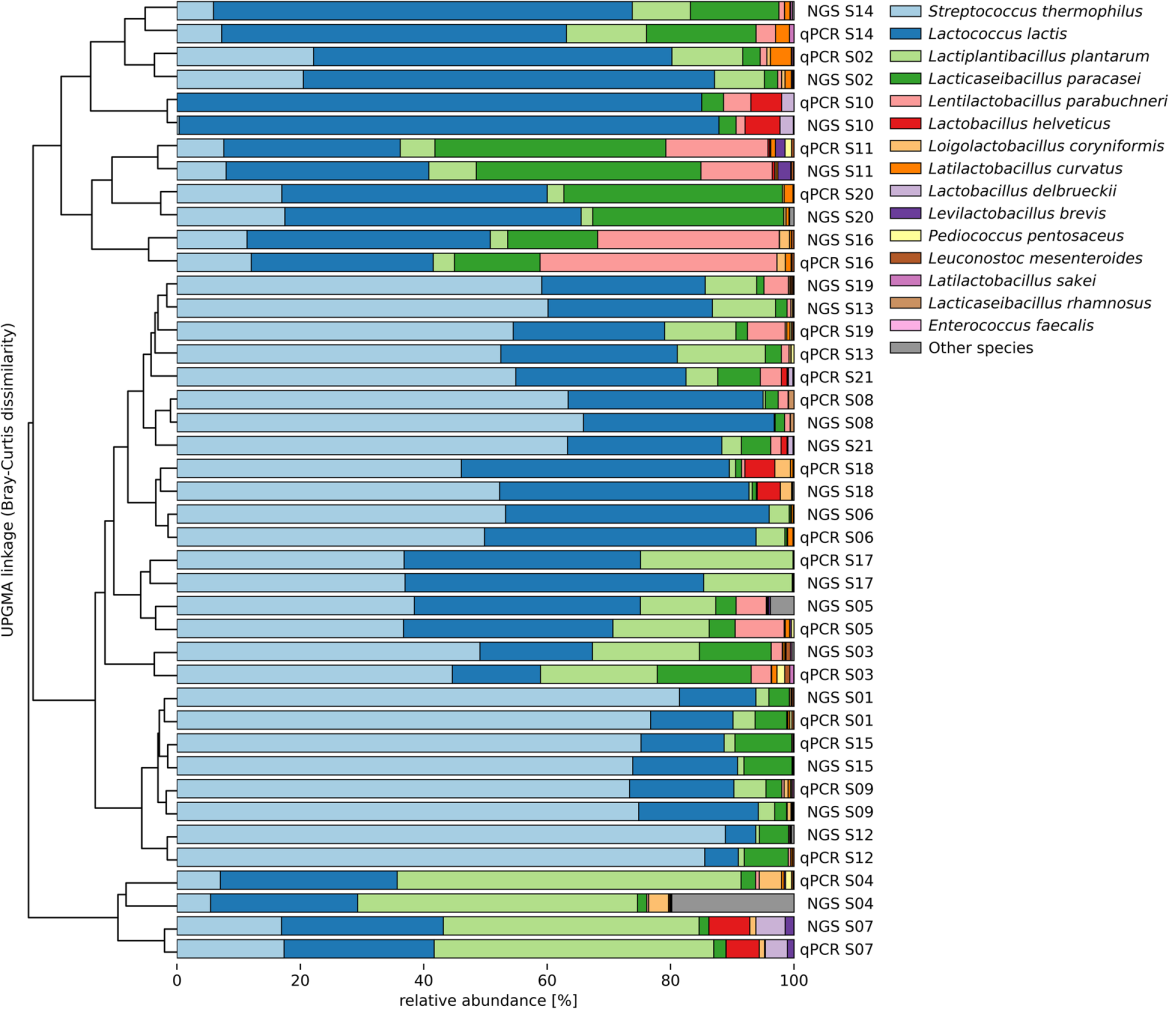
Comparison of HT-qPCR and 16S rRNA gene amplicon sequencing (NGS) data with corrections for *Lactiplantibacillus plantarum* group species and 16S rRNA gene copy normalization. Relative species compositions of the samples measured by HT-qPCR and NGS The samples are sorted and clustered according to the UPGMA linkage based on the Bray-Curtis dissimilarity. The relative abundance of the 15 species/groups detected by both methods are depicted with the species name; the other taxa were summarized as other species

The strength of the HT-qPCR approach lies in the fast and reliable analysis of samples with a known composition. The strength of NGS for exploratory purposes is very evident, as for example, bacterial species previously not associated to the cheese microbiota where discovered when NGS was applied to artisanal cheese samples [72]. Moreover here we show that overall, the results obtained by NGS and HT-qPCR mostly agreed for the relative abundance of a set of 15 shared bacterial species in 21 cheese DNA samples after the bioinformatics corrections for the *L. plantarum* group species. Unweighted pair group method with arithmetic mean (UPGMA) linkage based on Bray-Curtis dissimilarity clustered the measurements of NGS and HT-qPCR together for most of the 21 samples (Fig. 5). Only for cheese DNA samples S13, S19, and S21 did the results between the two methods diverge.

The present study aimed to evaluate the accuracy of a 16S rRNA gene amplicon sequencing approach in cheese by comparing it to absolute abundance data of selected taxa. During the early days of the use of 16S rRNA gene amplicon sequencing for the investigation of the cheese microbiota, the method was often compared to culture-dependent methods or qualitative culture-independent methods such as denaturing gradient gel electrophoresis [73–78]. However, advances in NGS technologies regarding optimization of the most discriminative 16S rRNA gene regions, primers, longer reads, and curated databases for specific ecosystems has increased the taxonomic resolution to the species level. Comparisons with quantitative culture-dependent methods are often limited in terms of species level resolution and bacteria in a viable but non-culturable state are not covered. Although HT-qPCR provided a limited coverage of the whole population in cheese, it has the ability to produce a more comprehensive and accurate evaluation with regard to the abundance of the selected bacterial species than previous culture-dependent and qualitative culture-independent approaches. On one hand, precise identification at the species level can be achieved, as with qualitative culture-independent methods, and on the other hand, absolute abundances can be measured.

Other approaches currently in use include comparisons between different protocols, sequencers, and analysis pipelines for evaluating new protocols or benchmarking [3, 79, 80]. These approaches address a variety of additional parameters, such as the performance of sequencing platforms, the influence of primer choice and library preparation protocols, as well as data analysis methods. These parameters were not within the scope of our study. Furthermore, there are aspects that are difficult to assess with these approaches, such as the influence of strain-specific variance in the number of copies of the rRNA operon or the species-specific PCR-associated bias [81]. Therefore, qPCR was particularly useful as a reference, because the influence of primer bias in qPCR reactions can be estimated accurately by the efficiency of the PCR reaction, and the variance of strain-specific 16S rRNA gene copy number per genome was bypassed by the selection of single copy gene targets.

A very detailed analysis is possible in our case because the microbial communities in the cheese core are quite well studied and shaped by the harsh and strictly controlled conditions during manufacturing and ripening [59, 82]. The HT-qPCR system used in this study was designed to cover frequent and abundant bacterial species in cooked, hard or semi-hard cheese with washed rinds made from raw milk. The data from 16S rRNA gene amplicon sequencing experiments was used to select appropriate target species to cover a high proportion of the most frequent and abundant bacterial community members. However, for other cheese types such as soft cheese varieties, the selection of the targets may be adapted. Likewise, the application of HT-qPCR to other fermented foods would be feasible. Since fermented foods such as sauerkraut or kimchi contain a core microbiota that partly overlaps with that of the cheese studied here [83, 84]. Nevertheless, this would most likely also require the development of some additional primer systems for species that are characteristic for the respective fermented food. For other, more complex ecosystems such as soil or the microbiota of specific human body sites, a comparison with the HT-qPCR approach is currently more challenging due to the high number of additional yet poorly defined taxa [85, 86].

Despite the identified bias for several species and the differences in 16S rRNA gene copy numbers, there was overall high agreement in relative abundances for the 15 species studied. This was an encouraging finding for the use of the NGS approach to study the microbiota of cheese.

A known, but sometimes neglected limitation of NGS approaches is that relative abundances are of limited use without knowledge of the absolute total abundance. Consequently, interpretation of the results may be challenging, especially for differential analyses or comparisons between samples with widely varying sampling or sequencing depths [19, 79, 87]. This is inherent in the method’s principle because in sequencing experiments, the number of counts does not reflect the underlying absolute number of molecules in the sample, but rather the ratio of counts per OTU or ASV multiplied by sequencing depth [87]. For the analysis of cheese and milk related samples this information can be relevant as illustrated in the following concrete example. First, one can consider a scenario in which two LAB species, e.g. *Lc. lactis* and *L. fermentum*, are present in a natural whey culture at relative abundances of 90 and 10%, respectively, which corresponds to an absolute abundance of for instance 10^7^ and 10^6^ copies/ml. After incubation, the ratio detected by NGS is 50 and 50%. This could be due to the growth of *L. fermentum* to 10^7^ copies/ml or to the reduction in the number of *Lc. lactis* to 10^6^ copies/ml due to autolysis or phage infection (while the growth of *L. fermentum* has stagnated). However, without knowing the total number of bacteria, distinguishing between the two scenarios is statistically more challenging and less accurate. In contrast, by using qPCR, we can measure the absolute abundance of the species directly without knowing the total number of bacteria.

As discussed above, we identified some flaws in taxa prediction accuracy using the selected NGS approach. Several studies have shown that the accurate identification of taxa depends on various factors such as the selected 16S rRNA gene region, read length, selected primers, sequencing platform, bioinformatics tools and reference databases [4, 7–9, 80, 88–90]. The approach for the bioinformatic analysis used in this study was already optimized by using an ecosystem-specific and manually curated reference database and bioinformatics algorithms with solid performance according to recent benchmarking studies [7, 89]. In the case of the already extensively discussed failure to differentiate the species of the *L. plantarum* group, the underlying cause was the high similarity of the 16S rRNA gene for these species. An improved prediction might only be achieved by longer reads or the selection of primers for a different target gene.

For NGS approaches, rarefaction curves are used to assess whether the sequencing depth is appropriate and if rare species/sequences could be identified with increasing sequencing depth [4]. However, the success to identify rare taxa and problems with a low sensitivity for certain taxa are often not only determined by the sequencing depth, the careful selection of the primers for the specific community under study is also important [90]. The low sensitivity for *P. pentosaceus* observed in the present study also indicates that species-specific PCR primer bias decreases the sensitivity significantly, even with an appropriate sequencing depth according to the saturation of the rarefaction curves (data not shown).

While qPCR is a well-established method with little potential for development beyond HT-qPCR, NGS is still experiencing a fast development. In this study, we applied single-end amplicon-based sequencing for the V1–V2 region of the rRNA gene with one analysis pipeline, including DAIRYdb and IDTAXA. Despite the limitations of NGS targeting only a small region, species annotation can be achieved thanks to highly curated databases [9]. In the future, amplicon free targeted sequencing by Nanopore can further improve the accuracy of NGS reducing the biases caused by preferential bindings of universal primers and allowing the sequencing of longer regions, such as the full 16S rRNA gene [1]. However, we also see future applications for HT-qPCR systems for the quantification of bacterial species in complex communities, such as those found in other fermented foods. The modular design of HT-qPCR and the possibility of the fully automated primer design pipeline SpeciesPrimer [91] strongly simplify the challenging process of primer design for a high-throughput system and we believe will facilitate the adaptation of HT-qPCR to ecosystems other than cheese.

## Conclusions

HT-qPCR and 16S rRNA gene amplicon sequencing provided highly comparable results for the qualitative and (semi-)quantitative characterization of bacterial communities in cheese. We have pointed out a number of differences and biases in measurements for several of the bacterial species included in this study. While the species assignments of most ASVs has been confirmed by HT-qPCR, we have also identified challenges in distinguishing *L. plantarum* from *L. pentosus* and in the correct assignment of *L. paraplantarum* based on the two variable regions of the 16S rRNA gene. Further, the different efficiencies for the measurement of several bacterial species were examined, and a potential PCR primer bias was identified.

We have highlighted the potential of NGS and HT-qPCR as complementary methods for both exploratory and screening purposes. NGS can be used to provide an overview of the microbial community, providing potential targets of interest for qPCR assay development, particularly in less known samples/environments. In return, qPCR can confirm species assignments, provide absolute quantitative data to better estimate the proportions of the bacterial composition, and draw attention to potential biases. HT-qPCR can then be used for more routine screening in environments with known bacterial composition.

Here, we demonstrated the application of NGS and HT-qPCR for the study of microbial communities in cheese and showed that the results were in substantial agreement. However, this approach may also be interesting for the study of the microbiota in other well-defined ecosystems.

## Supplementary Information

**Additional file 1.**

**Additional file 2.**

**Additional file 3.**

**Additional file 4.**

## Data Availability

The datasets generated and analyzed during the current study are available in the Sequence Read Archive (SRA) under the BioProject: PRJNA786903.
