# Supplementary material for: High-throughput qPCR and 16S rRNA gene amplicon sequencing as complementary methods for the investigation of the cheese microbiota
Source: BMC Microbiol. 2022 Feb 7;22:48. doi: 10.1186/s12866-022-02451-y (PMC8819918; doi:10.1186/s12866-022-02451-y)
Supplement: Supplementary file 1 — Additional file 1. [file 12866_2022_2451_MOESM1_ESM.zip › htqpcr_ngs_data-main/docs/htqpcr_ngs_comparison_R.html]

htqpcr\_ngs\_comparison\_R


GitHub repository

# High-throughput qPCR and 16S rRNA gene amplicon sequencing as complementary methods for the investigation of the cheese microbiota¶

## Data analysis¶

In our lab we are investigating the cheese microbiome and are interested in the influence of bacteria on cheese quality. Two different approaches were developed in recent years for the investigation of the cheese core microbiota. In this study we compared the results of the analysis of 21 Raclette du Valais PDO cheeses with two methods, high-throughput qPCR and 16S rRNA gene amplicon sequencing. We estimated the total bias of 16S rRNA gene amplicon sequencing (library preparation, sequencing and bioinformatics) using results from HT-qPCR as reference. Since we performed the analysis for both methods using the same DNA extractions the measured total bias is independent of the bias for the DNA extraction.

In [1]:

```
from IPython.display import HTML

HTML('''<script>
  code_show=false;
  function code_toggle() {
    if (code_show){
      $('div.input').hide();
    } else {
      $('div.input').show();
    }
    code_show = !code_show
  }
  $( document ).ready(code_toggle);
</script>
<form action="javascript:code_toggle()"><input type="submit" value="Toggle code on/off"></form>''')
```

Out[1]:

In [2]:

```
# Environment
import os
import json
import numpy as np
import pandas as pd
import seaborn as sns
import warnings
from pprint import pprint

from scipy.cluster.hierarchy import dendrogram, linkage
import matplotlib.pyplot as plt
from matplotlib import gridspec
from matplotlib.colors import ListedColormap

warnings.filterwarnings('ignore')

from comp_analysis import MathFunctions as mf
from comp_analysis import HelperFunctions as hf
from comp_analysis import PlotFunctions as pf

pd.set_option('display.max_rows', 1000)
pd.set_option('display.max_columns', 1000)
pd.set_option('display.width', 1000)
sns.set_context("paper")

# R environment
os.environ['R_HOME'] = os.path.expanduser('~/anaconda3/envs/htqpcr_ngs_comparison/lib/R')
os.environ['PKG_CONFIG_PATH'] = '/usr/lib/x86_64-linux-gnu/pkgconfig'

analysis_dir = "analysis_data"
figures_dir = "Figures"
dirs = [analysis_dir, figures_dir]
for d in dirs:
    if not os.path.isdir(d):
        os.mkdir(d)
      
%load_ext rpy2.ipython
```

In [3]:

```
%%R
R.Version()$version.string
```

```
[1] "R version 4.0.3 (2020-10-10)"
```

In [4]:

```
%%R
# Install R Packages the first time / load otherwise
if (!require('tidyverse')) install.packages('tidyverse'); library('tidyverse')
if (!require('devtools')) install.packages('devtools', dependencies=TRUE); library('devtools')
if (!require('phyloseq')) BiocManager::install("phyloseq"); library('phyloseq')
if (!require('metacal')) devtools::install_github("mikemc/metacal"); library('metacal')

# for problems installing the devtools package see:
# https://github.com/r-lib/devtools/issues/2131
```

```
R[write to console]: Loading required package: tidyverse

R[write to console]: ── Attaching packages ───────────────────────────────────────────────────────────────────────────────────────── tidyverse 1.3.1 ──

R[write to console]: ✔ ggplot2 3.3.3     ✔ purrr   0.3.4
✔ tibble  3.1.2     ✔ dplyr   1.0.6
✔ tidyr   1.1.3     ✔ stringr 1.4.0
✔ readr   1.4.0     ✔ forcats 0.5.1

R[write to console]: ── Conflicts ──────────────────────────────────────────────────────────────────────────────────────────── tidyverse_conflicts() ──
✖ dplyr::filter() masks stats::filter()
✖ dplyr::lag()    masks stats::lag()

R[write to console]: Loading required package: devtools

R[write to console]: Loading required package: usethis

R[write to console]: Loading required package: phyloseq

R[write to console]: Loading required package: metacal
```

---

### 16S rRNA gene amplicon sequencing¶

- Amplicons of the V1-V2 region of the 16S rRNA gene
- The analysis pipeline used the DAIRYdb database, Meola et al. 2019 for taxonomy assignment of the ASVs.
- A taxonomic resolution at species level was targeted

#### Summary amplicon sequencing raw data¶

In [5]:

```
# NGS raw data
raw_reads_df = pd.read_csv("rawdata/V18-22-21_ASV_counts_table.csv", header=0, index_col=0)

# filter very low reads
reads_df = raw_reads_df[
    (raw_reads_df.iloc[:, 9::].T.sum() > 2) & 
    (
        raw_reads_df.iloc[:, 9::].T.astype(bool).sum() 
        > 0.01 * len(raw_reads_df.iloc[:, 9::].T.index))]

# Add shorter labels
reads_df.columns = [
    "S" + n.split("-BC")[-1] if "-BC" in n else n 
    for n in reads_df.columns.tolist()]

# Extract data for Raclette du Valais samples
rac_reads = reads_df.iloc[:, :len(reads_df.columns) - 5]
rac_data = rac_reads[(rac_reads.iloc[:, 9:] != 0).any(axis=1)]

# reads info
hf.reads_info(rac_data, verbose=True)
# prints taxonomic data if verbose=True 
hf.tax_info(rac_data, verbose=False)
# create species table
ngs_count = hf().asv_to_speciestable(rac_data)
    
# Calculate relative abundance
ngs_reldna = 100 / ngs_count.sum() * ngs_count
ngs_reldna.replace(np.nan, 0, inplace=True)
filepath = os.path.join(analysis_dir, "ngs_rel_abundance.csv")
ngs_reldna.to_csv(filepath)

spec_mask = ngs_reldna.index.str.contains("Species")

fam = set(rac_data[rac_data["Genus"].str.contains("Genus")]["Genus"])
gen = set(rac_data[rac_data["Species"].str.contains("Species")]["Species"])
fam_num = [f.split("_")[0] for f in fam]
genus_num = [g for g in gen if g.split("_")[0] not in fam_num]
fam_num, genus_num

print("")
print("Level of assigned taxa")
print("Family:  ", len(fam_num))
print("Genus:   ", len(genus_num))                     
print("Species:", len(ngs_reldna[~spec_mask].index))
print("")
print("Samples:", ngs_reldna.shape[1])
```

```
Average sequencing depth: 471184 reads
Range: 361496-632269 reads
Total assigned reads: 9894860
Number of ASVs: 233

Level of assigned taxa
Family:   2
Genus:    0
Species: 45

Samples: 21
```

---

#### Additional data for Figure 1¶

In [6]:

```
# Show only species names for species present in more than 30% of the samples
tot_samples = ngs_reldna.shape[1]
prev_th = 0.3
# Split the plot for average reads
th = 0.5

prev_mask = (ngs_reldna.replace(0, np.nan).count(axis=1) >= tot_samples * prev_th)
prev = ngs_reldna.loc[prev_mask]
abund_mask = (prev.mean(axis=1) >= th)
high = prev.loc[prev.loc[abund_mask].mean(axis=1).sort_values(ascending=False).index]
low = prev.loc[prev.loc[~abund_mask].mean(axis=1).sort_values(ascending=False).index]
low.loc["Other sp.", :] = ngs_reldna.loc[~prev_mask].sum()
print("Number of species shown in plot (>30% prevalence):\n{}".format(len(prev.index)))
print("\nReads assigned to most prevalent species:\nAverage: {:.2f}% (range: {:.2f}-{:.0f}%)".format(
        prev.sum().mean(), prev.sum().min(), prev.sum().max()))

other_sp = ngs_reldna[ngs_reldna.loc[~prev_mask] != 0].count()
print("\nNumber of less prevalent species (Other sp.) per sample:")
print("\nAverage {:.2f} (range: {} - {})".format(other_sp.mean(), other_sp.min(), other_sp.max()))
pd.DataFrame(other_sp, columns=["Other sp."]).T
```

```
Number of species shown in plot (>30% prevalence):
21

Reads assigned to most prevalent species:
Average: 99.96% (range: 99.79-100%)

Number of less prevalent species (Other sp.) per sample:

Average 2.38 (range: 0 - 7)
```

Out[6]:

|  | S01 | S02 | S03 | S04 | S05 | S06 | S07 | S08 | S09 | S10 | S11 | S12 | S13 | S14 | S15 | S16 | S17 | S18 | S19 | S20 | S21 |
| --- | --- | --- | --- | --- | --- | --- | --- | --- | --- | --- | --- | --- | --- | --- | --- | --- | --- | --- | --- | --- | --- |
| Other sp. | 1 | 3 | 2 | 7 | 3 | 3 | 0 | 1 | 2 | 1 | 1 | 3 | 3 | 1 | 3 | 3 | 2 | 1 | 5 | 2 | 3 |

In [7]:

```
# Draw figure for NGS results
figsize=(8, 8)
sns.set_palette("Paired")
f, ax = plt.subplots(ncols=1, nrows=2, figsize=figsize, sharex=True, gridspec_kw={"hspace": 0.125})
pf.draw_ngs_barplot(high, low, ax, th)
save_to = os.path.join(figures_dir, "Figure_1.png")
plt.savefig(save_to, dpi=600, bbox_inches='tight')
plt.show()
```

##### Figure 1: Bacterial community composition determined by 16S rRNA gene amplicon sequencing¶

Species detected in Raclette du Valais PDO cheese DNA samples (S01-S21) with an average relative abundance above 0.5% are shown in the upper panel and the species with a lower average abundance in the lower panel. The relative abundance of the 21 species detected in more than 30% of the samples are depicted with the species name, the other 26 taxa were classified as other species.

---

### HT-qPCR¶

- The species targeted by the HT-qPCR system were selected based on preliminary data of Gruyère and Raclette du Valais 16S rRNA gene amplicon sequencing data.
- The primers for the HT-qPCR system were designed using SpeciesPrimer, Dreier et al. 2020.
- The script used to analyse the HT-qPCR data can be found on GitHub, Dreier et al. 2021.

In [8]:

```
%%capture
%run -i HTqPCR_dataparser/biomarkdataparser.py -i "rawdata/V18-22-21_htqpcr_rawdata.csv" \
--number 3 --replicates sample --outdir "analysis_data/htqpcrdata" --samplefilter 18-22-21 \
-x PW --transpose --datatoplot None
```

#### Summary HT-qPCR raw data¶

In [9]:

```
# read data and update labels
heatmap_file = os.path.join("analysis_data", "htqpcrdata", "copy_heatmap_data.csv")
df = pd.read_csv(heatmap_file, header=0, index_col=0)
df.columns = ["S"+n.split("EH ")[-1] for n in df.columns.values]
df.index = hf().new_assay_species_labels(df.index.values)
# remove all rows (species) that were not detected in DNA samples
pcr = df.dropna(how="all")
removed = df[~df.index.isin(pcr.index)]
print("Species not detected by qPCR in the samples:")
pprint(removed.index.tolist())

# number of copies (from log10 copies)
qpcr_count = round(10 ** pcr)
qpcr_count.loc["Lactococcus lactis"] = qpcr_count.loc[[
                                        "Lactococcus lactis subsp. lactis",
                                        "Lactococcus lactis subsp. cremoris"]].sum()
qpcr_count.drop([
    "Lactococcus lactis subsp. lactis",
    "Lactococcus lactis subsp. cremoris"], inplace=True)
qpcr_count = qpcr_count.replace(np.nan, 0).astype("int64")
filepath = os.path.join(analysis_dir, "qpcr_count.csv")
qpcr_count.to_csv(filepath)

qpcr_reldna = 100/qpcr_count.sum()*qpcr_count

print("")
print("qPCR dataset")
print("# Species: ", qpcr_reldna.shape[0])
print("# Samples:", qpcr_reldna.shape[1])
```

```
Species not detected by qPCR in the samples:
['Clostridium tyrobutyricum',
 'Enterococcus durans',
 'Enterococcus faecium',
 'Lacticaseibacillus casei',
 'Limosilactobacillus fermentum',
 'Pediococcus acidilactici',
 'Propionibacterium freudenreichii']

qPCR dataset
# Species:  16
# Samples: 21
```

---

In [10]:

```
# qPCR data heatmap - Only detected species sorted
species = list(pcr.index.values)
df = pd.read_csv("analysis_data/htqpcrdata/copy_annotation.csv", header=0, index_col=0)
df.columns = ["S"+n.split("EH ")[-1] for n in df.columns.values]
df.index = hf().new_assay_species_labels(df.index.values)

qpcrdata = hf.sort_sum(pcr)
annotation = df.loc[qpcrdata.index, qpcrdata.columns]

figsize=(12, 11)
f, ax = plt.subplots(ncols=1, nrows=1, figsize=figsize)
x , y, w, h = -0.065, 0.78, 0.03, 0.2
cax = f.add_axes([x, y, w, h])
pf.draw_qpcr_heatmap(qpcrdata, annotation, ax, cax)

save_to = os.path.join(figures_dir, "Figure_2.png")
plt.savefig(save_to, dpi=600, bbox_inches='tight')
```

##### Figure 1: Heatmap of HT-qPCR results¶

The heatmap annotation depicts the average log copies/µl and the standard deviation of technical triplicates. When not all samples were positive, the number of positive samples out of the total number of samples is given in brackets.

---

### Method comparison¶

- Both methods were applied to the same DNA samples
- HT-qPCR and amplicon sequencing are fundamentaly different in the results they produce.

  - Amplicon sequencing instruments have a fixed number of sequence reads and therefore the data are constraint to a constant sum (compositional) Gloor et al. 2017
  - HT-qPCR results consists of quantitative data for the targeted species when compared to a standard.

---

In [11]:

```
# Calculate stats for rel. abundance qPCR data
qpcr_stat = hf.get_stat_df(qpcr_reldna)
# Calculate stats for rel. abundance NGS data
ngs_comp = prev.copy()
ngs_comp.loc["Other sp.", :] = ngs_reldna.loc[~prev_mask].sum()
ngs_stat = hf.get_stat_df(ngs_comp)               
# Combine comp_stats for NGS and qPCR
comp_stats = pd.concat(
    [qpcr_stat[["Count", "Mean", "Std"]], ngs_stat[["Count", "Mean", "Std"]]], axis=1, sort=False)
# Add multiindex header
iterables = [['qPCR', 'NGS'], ["Count", "Avg. abundance [%]", "SD"]]
multiheader = pd.MultiIndex.from_product(iterables, names=['Method', 'Statistic'])
comp_stats.columns = multiheader
# Sort comp_stats
order = comp_stats[
        [("NGS", "Count"), ("qPCR", "Count")]
    ].sum(axis=1).sort_values(ascending=False).index
comp_stats = comp_stats.loc[order]
comp_stats = comp_stats.round(3)
comp_stats[
    [("NGS", "Count"), ("qPCR", "Count")]
        ] = comp_stats[
            [("NGS", "Count"), ("qPCR", "Count")]
                ].replace(np.nan, 0).astype('int64')

fp = os.path.join(figures_dir, "Table_1_data.csv")
comp_stats.to_csv(fp, index=True)
```

##### Table 1: Detected species and average relative abundance for the HT-qPCR and 16S rRNA gene amplicon sequencing (NGS) in 21 Raclette du Valais PDO cheese samples.¶

The relative abundance of the 21 species detected in more than 30% of the samples by NGS and L. paraplantarum exclusively detected by HT-qPCR are depicted with the species name, the other 26 taxa were classified as other species.

In [12]:

```
comp_stats
```

Out[12]:

| Method | qPCR | | | NGS | | |
| --- | --- | --- | --- | --- | --- | --- |
| Statistic | Count | Avg. abundance [%] | SD | Count | Avg. abundance [%] | SD |
| Lacticaseibacillus paracasei | 21 | 8.256 | 10.591 | 21 | 6.343 | 9.103 |
| Lactococcus lactis | 21 | 32.790 | 18.168 | 21 | 37.176 | 20.402 |
| Streptococcus thermophilus | 20 | 42.032 | 25.505 | 21 | 42.243 | 28.196 |
| Lactiplantibacillus plantarum | 20 | 12.001 | 14.762 | 20 | 3.466 | 6.557 |
| Loigolactobacillus coryniformis | 19 | 0.604 | 0.951 | 19 | 0.415 | 0.621 |
| Lentilactobacillus parabuchneri | 18 | 4.962 | 9.276 | 20 | 2.679 | 6.274 |
| Latilactobacillus curvatus | 18 | 0.767 | 0.854 | 19 | 0.263 | 0.302 |
| Lactobacillus helveticus | 5 | 3.278 | 2.476 | 21 | 0.632 | 1.473 |
| Leuconostoc mesenteroides | 9 | 0.306 | 0.219 | 14 | 0.128 | 0.147 |
| Lactobacillus delbrueckii | 4 | 1.583 | 1.537 | 19 | 0.658 | 1.888 |
| Levilactobacillus brevis | 7 | 0.471 | 0.610 | 16 | 0.262 | 0.545 |
| Latilactobacillus sakei | 10 | 0.242 | 0.247 | 12 | 0.098 | 0.120 |
| Pediococcus pentosaceus | 13 | 0.406 | 0.429 | 9 | 0.031 | 0.031 |
| Other sp. | 0 | NaN | NaN | 20 | 0.047 | 0.058 |
| Lactiplantibacillus pentosus | 0 | NaN | NaN | 20 | 5.210 | 7.905 |
| Lacticaseibacillus rhamnosus | 5 | 0.238 | 0.355 | 13 | 0.052 | 0.136 |
| Enterococcus gilvus | 0 | NaN | NaN | 15 | 0.030 | 0.045 |
| Lactiplantibacillus paraplantarum | 13 | 0.384 | 0.417 | 0 | NaN | NaN |
| Streptococcus uberis | 0 | NaN | NaN | 10 | 0.009 | 0.011 |
| Lentilactobacillus sunkii | 0 | NaN | NaN | 10 | 2.228 | 5.848 |
| Latilactobacillus fuchuensis | 0 | NaN | NaN | 9 | 0.021 | 0.024 |
| Enterococcus faecalis | 1 | 0.108 | NaN | 7 | 0.003 | 0.003 |
| Paucilactobacillus nenjiangensis | 0 | NaN | NaN | 8 | 0.143 | 0.198 |

---

In [13]:

```
# Create a dataframe with data in long format
cheese_df, shared_species = hf.create_summarydf(qpcr_count, ngs_count, qpcr_reldna, ngs_reldna)
# Identify commonly measured species (HT-qPCR and amplicon sequencing)
print(len(shared_species), "species shared between methods")
print()
shared_species
```

```
15 species shared between methods
```

Out[13]:

```
['Enterococcus faecalis',
 'Levilactobacillus brevis',
 'Loigolactobacillus coryniformis',
 'Latilactobacillus curvatus',
 'Lactobacillus delbrueckii',
 'Lactobacillus helveticus',
 'Lentilactobacillus parabuchneri',
 'Lacticaseibacillus paracasei',
 'Lactiplantibacillus plantarum',
 'Lacticaseibacillus rhamnosus',
 'Latilactobacillus sakei',
 'Leuconostoc mesenteroides',
 'Pediococcus pentosaceus',
 'Streptococcus thermophilus',
 'Lactococcus lactis']
```

In [14]:

```
# Plot
color_pal = sns.color_palette("colorblind")
fig, ax = plt.subplots(
    ncols=2, nrows=1, figsize = (11, 3.5), 
    gridspec_kw={"wspace": 0.25})

cat = {"shared_positive": 1, "qPCR_only": 2, "NGS_only": 4, "NGS_exclusive": 3}
pos_data = cheese_df.query('Category != "not_detected"')
pos_data["Sort"] = pos_data["Category"].map(cat)
plot_data = pos_data.sort_values(["Sort"])

pf.count_data_plot(plot_data, ax[0], color_pal)
pf.rel_data_plot(plot_data, ax[1], color_pal)

# Labels for plots
ax[0].text(
    -0.09, 0.98, 'A', fontsize=22, horizontalalignment='center',
     verticalalignment='center', transform=ax[0].transAxes)
ax[1].text(
    -0.09, 0.98, 'B', fontsize=22, horizontalalignment='center',
     verticalalignment='center', transform=ax[1].transAxes)
```

Out[14]:

```
Text(-0.09, 0.98, 'B')
```

##### Figure 3 AB: Comparison of count data and relative abundance data¶

---

In [15]:

```
# count data vs. rel. data
q = plot_data.query('Category == "qPCR_only"')
n = plot_data.query('Category == "NGS_only"')
print(
    "qPCR only (copies/ul) range: {:.0f} - {:.0f}".format(q["qPCR_count"].min(), q["qPCR_count"].max()))
print(
    " NGS only (reads)     range:    {:.0f} -  {:.0f}".format(n["NGS_count"].min(), n["NGS_count"].max()))
print()
print(
    "qPCR only [%] range: {:.4f} - {:.3f}".format(q["qPCR_rel"].min(), q["qPCR_rel"].max()))
print(
    " NGS only [%] range: {:.4f} - {:.3f}".format(n["NGS_rel"].min(), n["NGS_rel"].max()))

print("\nqPCR")
counts = q.groupby(["Species"])["Species"].count()

print("Lactiplantibacillus paraplantarum, {}x".format(counts[0]))
ql = q[q["Species"] == "Lactiplantibacillus paraplantarum"]
print(
    "qPCR only (copies/ul) range: {:.0f} - {:.0f}".format(ql["qPCR_count"].min(), ql["qPCR_count"].max()))
print(
    "qPCR only [%] range: {:.4f} - {:.3f}".format(ql["qPCR_rel"].min(), ql["qPCR_rel"].max()))

print("\nPediococcus pentosaceus, {}x".format(counts[1]))
qp = q[q["Species"] == "Pediococcus pentosaceus"]
print(
    "qPCR only (copies/ul) range: {:.0f} - {:.0f}".format(qp["qPCR_count"].min(), qp["qPCR_count"].max()))
print(
    "qPCR only [%] range: {:.4f} - {:.3f}".format(qp["qPCR_rel"].min(), qp["qPCR_rel"].max()))

print("\nNGS")
ngs_df = pd.DataFrame()
ngs_grouped = n.groupby(["Species"])
ngs_df["Count"] = ngs_grouped["Species"].count()
ngs_df["reads (min)"] = ngs_grouped["NGS_count"].min().astype("int64")
ngs_df["reads (max)"] = ngs_grouped["NGS_count"].max().astype("int64")
ngs_df["% (min)"] = ngs_grouped["NGS_rel"].min().round(4)
ngs_df["% (max)"] = ngs_grouped["NGS_rel"].max().round(4)
pprint(ngs_df.sort_values(["reads (max)"], ascending=False))
```

```
qPCR only (copies/ul) range: 1389 - 53306
 NGS only (reads)     range:    1 -  1369

qPCR only [%] range: 0.0141 - 1.326
 NGS only [%] range: 0.0002 - 0.322

qPCR
Lactiplantibacillus paraplantarum, 13x
qPCR only (copies/ul) range: 1472 - 53306
qPCR only [%] range: 0.0264 - 1.326

Pediococcus pentosaceus, 5x
qPCR only (copies/ul) range: 1389 - 6542
qPCR only [%] range: 0.0141 - 0.239

NGS
                                 Count  reads (min)  reads (max)  % (min)  % (max)
Species                                                                           
Streptococcus thermophilus           1         1369         1369   0.3219   0.3219
Levilactobacillus brevis             9            5          643   0.0010   0.1135
Lactobacillus helveticus            16           21          155   0.0044   0.0429
Lactobacillus delbrueckii           15           13          117   0.0021   0.0251
Lentilactobacillus parabuchneri      2           16           54   0.0030   0.0095
Latilactobacillus curvatus           1           49           49   0.0086   0.0086
Latilactobacillus sakei              2           42           49   0.0088   0.0114
Leuconostoc mesenteroides            5           14           42   0.0035   0.0100
Lacticaseibacillus rhamnosus         8            1           40   0.0002   0.0091
Pediococcus pentosaceus              1           26           26   0.0055   0.0055
Enterococcus faecalis                6            2           12   0.0005   0.0033
```

#### Differences in detection of species¶

- Species that were only detected by HT-qPCR in certain samples were *Lactiplantibacillus paraplantarum* and *Pediococcus pentosaceus*.

  - *Lactiplantibacillus paraplantarum* was measured 13 times by HT-qPCR with copies/ul in a range between 1472 and 53306 copies/ul.
  - *Lactiplantibacillus paraplantarum* was never detected by amplicon sequencing (Table 1).
  - *Pediococcus pentosaceus* was measured in 5 samples by HT-qPCR, but not by amplicon sequencing with a range of 1389 - 6542 copies/ul.

- 11 species, also covered by HT-qPCR assays, were only detected by amplicon sequencing. The 66 measurements were in a range between 1 and 1369 reads and had a relative abundance between 0.0002 and 0.322%.

---

In [16]:

```
# shared species
mask = ngs_reldna.index.isin(shared_species)
# not targeted by qPCR but abundant, separate the two most abundant
highest_abund = ngs_reldna[~mask].sum(axis=1).nlargest(2).index
abund_mask = ngs_reldna.index.isin(highest_abund)
# remaining
other_mask = (~mask) & (~abund_mask)
# Table data
tbl = pd.DataFrame()
tbl["Shared species [%]"] = ngs_reldna[mask].sum()
tbl["Shared species [n]"] = ngs_reldna[ngs_reldna[mask] !=0].count()
tbl["Other species [%]"] = ngs_reldna[other_mask].sum()
tbl["Other species [n]"] = ngs_reldna[ngs_reldna[other_mask] !=0].count()
tbl[highest_abund] = ngs_reldna.loc[highest_abund].T

# Create table labels
tbl.index.name = "Sample"
iterables = [['Shared species', 'Other species'], ["[%]", "[n]"]]
m1 = pd.MultiIndex.from_product(iterables)
m2 = pd.MultiIndex.from_tuples(tuple(m1) + tuple([(i, "[%]") for i in tbl.columns[-2::]]))
tbl.columns = m2
tbl = tbl.round(2).replace(0, np.nan)
save_to = os.path.join(figures_dir, "Table_2_data.csv")
tbl.to_csv(save_to)

mean = tbl[("Shared species", "[%]")].mean().round(2)
mini = tbl[("Shared species", "[%]")].min().round(2)
maxi = tbl[("Shared species", "[%]")].max().round(2)

# Samples with below average coverage
print("shared species")
print("Avg. {}, (range: {}-{})".format(mean, mini, maxi))
print("\nSamples with below average coverage")
print(tbl[tbl[("Shared species", "[%]")] < mean])

mean = tbl[("Lactiplantibacillus pentosus", "[%]")].mean().round(2)
mini = tbl[("Lactiplantibacillus pentosus", "[%]")].min().round(2)
maxi = tbl[("Lactiplantibacillus pentosus", "[%]")].max().round(2)

# Samples with above-average rel. abundance of L. pentosus
print()
print("Samples with above-average rel. abundance of L. pentosus")
print("Avg. {}, (range: {}-{})".format(mean, mini, maxi))
print(tbl[tbl[("Lactiplantibacillus pentosus", "[%]")] > mean].index.to_list())

mean = tbl[("Lentilactobacillus sunkii", "[%]")].mean().round(2)
mini = tbl[("Lentilactobacillus sunkii", "[%]")].min().round(2)
maxi = tbl[("Lentilactobacillus sunkii", "[%]")].max().round(2)

# Samples with above-average rel. abundance of L. sunkii
print()
print("Samples with above-average rel. abundance of L. sunkii")
print("Avg. {}, (range: {}-{})".format(mean, mini, maxi))
print(tbl[tbl[("Lentilactobacillus sunkii", "[%]")] > mean].index.to_list())
print()

mean = tbl[("Other species", "[%]")].mean().round(2)
mini = tbl[("Other species", "[%]")].min().round(2)
maxi = tbl[("Other species", "[%]")].max().round(2)

print("other species")
print("Avg. {}, (range: {}-{})".format(mean, mini, maxi))
```

```
shared species
Avg. 93.84, (range: 44.69-99.93)

Samples with below average coverage
       Shared species     Other species     Lactiplantibacillus pentosus Lentilactobacillus sunkii
                  [%] [n]           [%] [n]                          [%]                       [%]
Sample                                                                                            
S03             91.16  14          0.36   6                         8.47                       NaN
S04             44.69  14          0.44  11                        36.27                     18.60
S05             90.24  13          0.07   4                         6.33                      3.36
S07             92.47  12          0.01   1                         7.52                       NaN
S17             89.88  11          0.04   2                        10.08                       NaN

Samples with above-average rel. abundance of L. pentosus
Avg. 5.21, (range: 0.05-36.27)
['S03', 'S04', 'S05', 'S07', 'S11', 'S13', 'S17']

Samples with above-average rel. abundance of L. sunkii
Avg. 3.71, (range: 0.01-18.6)
['S04']

other species
Avg. 0.13, (range: 0.01-0.63)
```

##### Table 2: Relative abundance data of species detected by 16S rRNA gene amplicon sequencing.¶

Percentage of reads assigned to species also covered by HT-qPCR (shared) and other species that were not covered by HT-qPCR. Two species *L. pentosus* and *L. sunkii* with the largest overall abundance are shown separately.

In [17]:

```
tbl
```

Out[17]:

|  | Shared species | | Other species | | Lactiplantibacillus pentosus | Lentilactobacillus sunkii |
| --- | --- | --- | --- | --- | --- | --- |
|  | [%] | [n] | [%] | [n] | [%] | [%] |
| Sample |  |  |  |  |  |  |
| S01 | 98.73 | 13 | 0.03 | 3 | 1.24 | 0.01 |
| S02 | 95.06 | 12 | 0.09 | 6 | 4.83 | 0.02 |
| S03 | 91.16 | 14 | 0.36 | 6 | 8.47 | NaN |
| S04 | 44.69 | 14 | 0.44 | 11 | 36.27 | 18.60 |
| S05 | 90.24 | 13 | 0.07 | 4 | 6.33 | 3.36 |
| S06 | 98.62 | 12 | 0.01 | 5 | 1.37 | NaN |
| S07 | 92.47 | 12 | 0.01 | 1 | 7.52 | NaN |
| S08 | 99.93 | 10 | 0.02 | 3 | 0.05 | NaN |
| S09 | 98.89 | 12 | 0.11 | 3 | 1.00 | NaN |
| S10 | 99.91 | 7 | 0.09 | 1 | NaN | NaN |
| S11 | 94.59 | 15 | 0.05 | 3 | 5.36 | NaN |
| S12 | 99.31 | 13 | 0.09 | 5 | 0.33 | 0.27 |
| S13 | 94.06 | 12 | 0.14 | 4 | 5.81 | NaN |
| S14 | 94.76 | 13 | 0.23 | 5 | 5.01 | NaN |
| S15 | 99.26 | 10 | 0.03 | 5 | 0.71 | NaN |
| S16 | 98.11 | 11 | 0.02 | 4 | 1.86 | NaN |
| S17 | 89.88 | 11 | 0.04 | 2 | 10.08 | NaN |
| S18 | 99.50 | 11 | 0.18 | 3 | 0.31 | NaN |
| S19 | 95.44 | 14 | 0.13 | 6 | 4.43 | NaN |
| S20 | 98.24 | 10 | 0.63 | 5 | 1.12 | 0.01 |
| S21 | 97.85 | 13 | 0.03 | 7 | 2.11 | NaN |

---

## Bias estimation¶

McLaren et al., 2019 developed a model to estimate the bias in metagenomic sequencing experiments. We used their tool metacal to estimate the bias of the amplicon sequencing approach using HT-qPCR data as reference.

- The same samples (DNA extractions) were used for the measurements with both methods.
  - ~~Extraction bias~~
- Potential bias/systematic errors
  - PCR bias
    - primer binding
    - amplification efficiency
    - variation in target gene copy number
  - Sequencing bias
  - Bioinformatics bias
    - binning
    - database

Both methods have the potential for systematic error or bias, however for HT-qPCR we can estimate the amplification efficiency using qPCR standards with which also the copies/ul are calculated. For the qPCR assays single copy genes were selected and the number of copies was checked with publicly available genomes. Therefore we selected qPCR as the reference method and and make the simplified assumption that this method has no bias for the following comparison. We made further simplifications, for example we only compared the abundance of species measured by both methods.

---

In [18]:

```
# Find shared species between the methods
shared_species = sorted(list(set(qpcr_count.index) & set(ngs_count.index)), reverse=True)
samples = list(qpcr_count.columns)
# keep only the data for these species (order aligned)
shared_qpcr = qpcr_count.loc[shared_species, samples]
shared_ngs = ngs_count.loc[shared_species, samples]
assert shared_qpcr.shape == shared_ngs.shape

# use pseudocounts (=1) for the observed data for species detected by HT-qPCR (reference) 
not_observed = (shared_ngs == 0) & (shared_qpcr != 0) 
shared_ngs[not_observed] = 1

# remove data points that are only in the observed data (metacal would also remove these automatically) 
not_ref = (shared_ngs != 0) & (shared_qpcr == 0)
shared_ngs[not_ref] = 0

ref_df = shared_qpcr.copy()
obs_df = shared_ngs.copy()

# Import dataframes to R
%R -i ref_df -i obs_df
```

In [19]:

```
%%R
# Bias estimation
reference <- ref_df %>% as("matrix")
observed  <- obs_df %>% as("matrix")

stopifnot(identical(dimnames(observed), dimnames(reference)))

bias_fit <- estimate_bias(
  observed,
  reference, 
  margin = 2, # samples as columns
  boot = TRUE
)

bias_fit.summary <- summary(bias_fit)
bias_table <- bias_fit.summary$coefficients

print(bias_fit.summary)
```

```
Summary of a metacal bias fit.

Estimated relative efficiencies:
# A tibble: 15 x 4
   taxon                           estimate gm_mean gm_se
   <chr>                              <dbl>   <dbl> <dbl>
 1 Streptococcus thermophilus        2.33    2.33    1.05
 2 Pediococcus pentosaceus           0.0420  0.0423  1.48
 3 Loigolactobacillus coryniformis   1.60    1.60    1.05
 4 Levilactobacillus brevis          2.47    2.46    1.11
 5 Leuconostoc mesenteroides         1.36    1.35    1.09
 6 Lentilactobacillus parabuchneri   0.918   0.917   1.07
 7 Latilactobacillus sakei           0.985   0.985   1.11
 8 Latilactobacillus curvatus        0.722   0.720   1.09
 9 Lactococcus lactis                2.57    2.57    1.05
10 Lactobacillus helveticus          1.85    1.86    1.07
11 Lactobacillus delbrueckii         4.05    4.07    1.08
12 Lactiplantibacillus plantarum     0.499   0.499   1.10
13 Lacticaseibacillus rhamnosus      0.987   0.982   1.20
14 Lacticaseibacillus paracasei      1.57    1.57    1.05
15 Enterococcus faecalis             0.196   0.196   1.03

Geometric standard error estimated from 1000 bootstrap replicates.
```

In [20]:

```
# Export bias_table from R
%R -o bias_table

biasdf = bias_table.copy()
biasdf["errorbar_min"] = biasdf["estimate"] / (biasdf["gm_se"] ** 2)
biasdf["errorbar_max"] = biasdf["estimate"] * (biasdf["gm_se"] ** 2)
```

In [21]:

```
# Plot
color_pal = sns.color_palette("colorblind")

fig = plt.figure(figsize = (11, 7))
ax = gridspec.GridSpec(ncols=2, nrows=2, figure=fig, hspace=0.4, wspace=0.5, width_ratios=[1, 0.85])

f_ax1 = fig.add_subplot(ax[0, 0])
f_ax2 = fig.add_subplot(ax[1, 0])
f_label = fig.add_subplot(ax[0, 1])
f_label.axis("off")
f_ax3 = fig.add_subplot(ax[0:2,1])

cat = {"shared_positive": 1, "qPCR_only": 2, "NGS_only": 4, "NGS_exclusive": 3}
pos_data = cheese_df.query('Category != "not_detected"')
pos_data["Sort"] = pos_data["Category"].map(cat)
plot_data = pos_data.sort_values(["Sort"])

pf.count_data_plot(plot_data, f_ax1, color_pal)
pf.rel_data_plot(plot_data, f_ax2, color_pal)

pf.bias_plot(biasdf, ax=f_ax3, colors=["black"])
f_ax3.set_xticks([0.1, 0.3, 0.5, 1, 2, 3])
f_ax3.set_xticklabels([0.1, 0.3, 0.5, 1, 2, 3])
ylim = f_ax3.get_ylim()
f_ax3.set_ylim(0.5, 15.5)

f_ax3.tick_params(length=0, which="minor")
f_ax3.grid("on", color="white")
f_ax3.set_facecolor('gainsboro')
for spine in f_ax3.spines:
    f_ax3.spines[spine].set_color("white")

# Labels for plots
f_ax1.text(
    -0.09, 0.95, 'A', fontsize=22, horizontalalignment='center',
     verticalalignment='bottom', transform=f_ax1.transAxes)
f_ax2.text(
    -0.09, 0.95, 'B', fontsize=22, horizontalalignment='center',
     verticalalignment='bottom', transform=f_ax2.transAxes)
f_label.text(
    -0.4, 0.95, 'C', fontsize=22, horizontalalignment='center',
     verticalalignment='bottom', transform=f_label.transAxes)

save_to = os.path.join(figures_dir, "Figure_3.png")
plt.savefig(save_to, dpi=600, bbox_inches='tight')
```

##### Figure 3: Comparison of HT-qPCR and 16S rRNA gene amplicon sequencing (NGS)¶

A) Logarithmic HT-qPCR data (y-axis) and logarithmic count data corresponding to the NGS approach (x-axis). The red line depicts the threshold of 800 copies/µl used for the HT-qPCR data analysis. Shared positive: Measurements obtained with both methods. qPCR only/NGS only: Measurements obtained solely by one of the two methods, HT-qPCR or NGS, respectively. NGS exclusive: Measurements of taxa by NGS for which no HT-qPCR assay was available. The number of observations for each group is given in brackets. B) Direct comparison of the relative abundance data of HT-qPCR (copies/µl) on the y-axis and NGS (reads) on the x-axis. The taxa that were only detected by NGS (NGS exclusive) were not considered. C) Plot of the bias point estimates ± two geometric standard errors calculated for the NGS approach using the HT-qPCR approach as reference method. Only the data of the shared positive measurements was used for the bias estimation.

#### Correction for *L. plantarum* group species (See Supplementary Figure S1)¶

We have observed a large bias for several species (Figure 3C). The most negatively biased were *L. plantarum*, *E. faecalis* and *P. pentosaceus*.

- It is known that the species of th *L. plantarum* group are difficult to differentiate based on 16S rRNA gene sequences. Therefore we investigated the 16S rRNA gene region (V1-V2) of reference genomes for *L. plantarum*, *L. paraplantarum* and *L. pentosus*. Multiple sequence alignments of the V1-V2 region have shown that the amplicon sequences of *L. plantarum* and *L. pentosus* were nearly identical and that the sequence of *L. paraplantarum* had only few different nucleotides. An alignment of the ASVs (amplicon sequence variants) observed for *L. plantarum* and *L. pentosus* and the reference sequences from reference genomes and the DAIRYdb has shown that sequences from *L. paraplantarum* formed a separate cluster. However, due to the highly similar reference sequences in the DAIRYdb these ASVs were assigned to either *L. plantarum* or *L. pentosus*. Since these three species were not separated by the analysis pipeline we applied a correction by grouping the reads together in an *L. plantarum* group for further analysis of the data.

##### Supplementary Figure S1: Analysis of the potential to differentiate L. plantarum group species based on V1-V2 variable regions of the 16S rRNA gene.¶

A) Alignment of the V1-V2 16S rRNA gene region of the representative genomes of *L. plantarum*, *L. paraplantarum* and *L. pentosus*.
B) Alignment of the DAIRYdb 16S rRNA gene reference sequences.
C) The best-scoring maximum likelihood tree from alignments of the V1-V2 16S rRNA gene sequences including the representative genomes, the DAIRYdb reference sequences and the ASVs for *L. plantarum*, *L. paraplantarum* and *L. pentosus*.

---

#### Supplementary Figure S2: *L. plantarum* group discrimination of 16S rRNA gene amplicon sequencing targeting the V3-V4 region¶

---

- An multiple sequence alignment (MSA) of the V1-V2 region of the 16S rRNA gene for representative genomes from NCBI for these species has shown that *P. pentosaceus* harbors a single nucleotide difference in the forward primer binding region (**Supplementary Figure S2**). This could affect the amplification efficiency of these amplicons and the reason for the strong bias for this taxon in the results.

- *E. faecalis* was only once detected by HT-qPCR and seven times by amplicon sequencing. The difference in the number of detections seems reasonable since the technical limit of detection for qPCR is around 800 copies/ul and the relative abundance of *E. faecalis* was very low for these six samples. The MSA did not show any indications for issues with primer binding.

In [22]:

```
# For Enterocccus faecalis only one shared measurement was available
cheese_df.query('Species == "Enterococcus faecalis" & NGS_rel > 0')
```

Out[22]:

|  | Sample | Species | qPCR\_count | NGS\_count | qPCR\_rel | NGS\_rel | Category |
| --- | --- | --- | --- | --- | --- | --- | --- |
| 11 | S02 | Enterococcus faecalis | 3071.0 | 40.0 | 0.107748 | 0.009505 | shared\_positive |
| 211 | S04 | Enterococcus faecalis | 0.0 | 5.0 | 0.000000 | 0.000963 | NGS\_only |
| 214 | S05 | Enterococcus faecalis | 0.0 | 12.0 | 0.000000 | 0.003320 | NGS\_only |
| 230 | S11 | Enterococcus faecalis | 0.0 | 2.0 | 0.000000 | 0.000542 | NGS\_only |
| 234 | S12 | Enterococcus faecalis | 0.0 | 4.0 | 0.000000 | 0.000633 | NGS\_only |
| 242 | S14 | Enterococcus faecalis | 0.0 | 10.0 | 0.000000 | 0.002126 | NGS\_only |
| 260 | S19 | Enterococcus faecalis | 0.0 | 2.0 | 0.000000 | 0.000516 | NGS\_only |

##### Supplementary Figure S3: Alignment of the 16S rRNA gene primer binding regions.¶

---

## Bioinformatics bias correction¶

#### 16S rRNA gene copy number correction¶

Another potential source of PCR bias for amplicon sequencing may be the variation (inter- or intra-species) in the number of copies of the 16S rRNA gene.
The qPCR assays were designed to target single copy genes.

We estimated the average copy numbers of the taxons and performed copy number normalization

16S copy number data was extracted from the rrnDB, (Stoddard et al.)

In [23]:

```
average_16S_CN = 5.29 # Starke et al. 2020, https://doi.org/10.1007/s00248-020-01586-7
specieslist = list(set(list(qpcr_count.index.values) + list(ngs_count.index.values)))

inputfile = 'rawdata/rrnDB-5.7_pantaxa_stats_NCBI.tsv' # you may want to update this filename for newer version of the database

if os.path.isfile(inputfile):
    copy_tbl, copy_dict = hf.find_16S_copy_numbers(inputfile, specieslist, average_16S_CN)  
else:
    copy_tbl = pd.read_csv("rawdata/16S_copy_number_table.csv", index_col=0, header=0)
    with open("rawdata/copydict.json", "r") as f:
        for line in f:
            copy_dict = json.loads(line)

copy_tbl.to_csv("Figures/Supplementary_Table_S1_data.csv")

cn_bias_dict = dict(mf.center_elts(pd.DataFrame.from_dict(copy_dict, orient='index'))[0])
```

##### Supplementary Table S1: 16S rRNA gene copy numbers for taxa detected in Raclette du Valais PDO cheeses¶

In [24]:

```
copy_tbl.loc[shared_species, :]
```

Out[24]:

|  | Rank | N | Min. | Max. | Median | Avg. | SD |
| --- | --- | --- | --- | --- | --- | --- | --- |
| Streptococcus thermophilus | species | 66.0 | 5.0 | 6.0 | 6.0 | 5.67 | 0.47 |
| Pediococcus pentosaceus | species | 18.0 | 5.0 | 5.0 | 5.0 | 5.00 | 0.00 |
| Loigolactobacillus coryniformis | species | 4.0 | 1.0 | 5.0 | 5.0 | 4.00 | 1.73 |
| Levilactobacillus brevis | species | 18.0 | 4.0 | 6.0 | 5.0 | 5.00 | 0.33 |
| Leuconostoc mesenteroides | species | 20.0 | 3.0 | 4.0 | 4.0 | 3.95 | 0.22 |
| Lentilactobacillus parabuchneri | species | 3.0 | 5.0 | 5.0 | 5.0 | 5.00 | 0.00 |
| Latilactobacillus sakei | species | 20.0 | 5.0 | 8.0 | 7.0 | 6.85 | 0.65 |
| Latilactobacillus curvatus | species | 12.0 | 6.0 | 6.0 | 6.0 | 6.00 | 0.00 |
| Lactococcus lactis | species | 48.0 | 5.0 | 6.0 | 6.0 | 5.94 | 0.24 |
| Lactobacillus helveticus | species | 17.0 | 4.0 | 5.0 | 4.0 | 4.24 | 0.42 |
| Lactobacillus delbrueckii | species | 25.0 | 8.0 | 11.0 | 9.0 | 8.92 | 0.63 |
| Lactiplantibacillus plantarum | species | 135.0 | 1.0 | 5.0 | 5.0 | 4.93 | 0.50 |
| Lacticaseibacillus rhamnosus | species | 26.0 | 5.0 | 5.0 | 5.0 | 5.00 | 0.00 |
| Lacticaseibacillus paracasei | species | 44.0 | 5.0 | 5.0 | 5.0 | 5.00 | 0.00 |
| Enterococcus faecalis | species | 50.0 | 4.0 | 4.0 | 4.0 | 4.00 | 0.00 |

In [25]:

```
# Correction of the bioinformatics analysis bias of L. plantarum group species 
corr_ngs_count = ngs_count.copy()
corr_ngs_count.loc["Lactiplantibacillus plantarum", :] = corr_ngs_count.loc[
    ["Lactiplantibacillus plantarum", "Lactiplantibacillus pentosus"]].sum()
corr_ngs_count = corr_ngs_count.drop(
    ["Lactiplantibacillus pentosus"])
corr_ngs_rel = mf.calc_rel_abund(corr_ngs_count)

corr_qpcr_count = qpcr_count.copy()
corr_qpcr_count.loc["Lactiplantibacillus plantarum", :] = corr_qpcr_count.loc[
    ["Lactiplantibacillus plantarum", "Lactiplantibacillus paraplantarum"]].sum()
corr_qpcr_count = corr_qpcr_count.drop(
    ["Lactiplantibacillus paraplantarum"])
corr_qpcr_rel = mf.calc_rel_abund(corr_qpcr_count)

corr_cheese_df, corr_shared_species = hf.create_summarydf(
    corr_qpcr_count, corr_ngs_count, corr_qpcr_rel, corr_ngs_rel)
```

In [26]:

```
# shared species
mask = corr_ngs_rel.index.isin(corr_shared_species)
# not targeted by qPCR but high abundance
highest_abund = corr_ngs_rel[~mask].sum(axis=1).nlargest(1).index
abund_mask = corr_ngs_rel.index.isin(highest_abund)
# remaining
other_mask = (~mask) & (~abund_mask)
# Table data
corr_tbl = pd.DataFrame()
corr_tbl["Shared species [%]"] = corr_ngs_rel[mask].sum()
corr_tbl["Shared species [n]"] = corr_ngs_rel[corr_ngs_rel[mask] !=0].count()
corr_tbl["Other species [%]"] = corr_ngs_rel[other_mask].sum()
corr_tbl["Other species [n]"] = corr_ngs_rel[corr_ngs_rel[other_mask] !=0].count()
corr_tbl[highest_abund] = corr_ngs_rel.loc[highest_abund].T

corr_tbl.index.name = "Sample"
iterables = [['Shared species', 'Other species'], ["[%]", "[n]"]]
m1 = pd.MultiIndex.from_product(iterables)
m2 = pd.MultiIndex.from_tuples(tuple(m1) + tuple([(i, "[%]") for i in corr_tbl.columns[-1::]]))
corr_tbl.columns = m2
corr_tbl = corr_tbl.round(2).replace(0, np.nan)
save_to = os.path.join(figures_dir, "Supplementary_Table_S2_data.csv")
corr_tbl.to_csv(save_to)
```

##### Supplementary Table S2: Relative abundance data of species detected by 16S rRNA gene amplicon sequencing after L. plantarum group correction.¶

In [27]:

```
corr_tbl
```

Out[27]:

|  | Shared species | | Other species | | Lentilactobacillus sunkii |
| --- | --- | --- | --- | --- | --- |
|  | [%] | [n] | [%] | [n] | [%] |
| Sample |  |  |  |  |  |
| S01 | 99.96 | 13 | 0.03 | 3 | 0.01 |
| S02 | 99.89 | 12 | 0.09 | 6 | 0.02 |
| S03 | 99.64 | 14 | 0.36 | 6 | NaN |
| S04 | 80.96 | 14 | 0.44 | 11 | 18.60 |
| S05 | 96.57 | 13 | 0.07 | 4 | 3.36 |
| S06 | 99.99 | 12 | 0.01 | 5 | NaN |
| S07 | 99.99 | 12 | 0.01 | 1 | NaN |
| S08 | 99.98 | 10 | 0.02 | 3 | NaN |
| S09 | 99.89 | 12 | 0.11 | 3 | NaN |
| S10 | 99.91 | 7 | 0.09 | 1 | NaN |
| S11 | 99.95 | 15 | 0.05 | 3 | NaN |
| S12 | 99.64 | 13 | 0.09 | 5 | 0.27 |
| S13 | 99.86 | 12 | 0.14 | 4 | NaN |
| S14 | 99.76 | 13 | 0.23 | 5 | NaN |
| S15 | 99.97 | 10 | 0.03 | 5 | NaN |
| S16 | 99.97 | 11 | 0.02 | 4 | NaN |
| S17 | 99.96 | 11 | 0.04 | 2 | NaN |
| S18 | 99.82 | 11 | 0.18 | 3 | NaN |
| S19 | 99.87 | 14 | 0.13 | 6 | NaN |
| S20 | 99.36 | 10 | 0.63 | 5 | 0.01 |
| S21 | 99.97 | 13 | 0.03 | 7 | NaN |

In [28]:

```
# Coverage after correction for L. plantarum group
mean = corr_tbl[("Shared species", "[%]")].mean().round(2)
mini = corr_tbl[("Shared species", "[%]")].min().round(2)
maxi = corr_tbl[("Shared species", "[%]")].max().round(2)

print("shared species")
print("Avg. {}, (range: {}-{})".format(mean, mini, maxi))
print("\nSamples with below average coverage\n")
print(corr_tbl[corr_tbl[("Shared species", "[%]")] < mean])
```

```
shared species
Avg. 98.81, (range: 80.96-99.99)

Samples with below average coverage

       Shared species     Other species     Lentilactobacillus sunkii
                  [%] [n]           [%] [n]                       [%]
Sample                                                               
S04             80.96  14          0.44  11                     18.60
S05             96.57  13          0.07   4                      3.36
```

In [29]:

```
# Find shared species between the methods for corrected data
shared_species = sorted(list(set(corr_qpcr_count.index) & set(corr_ngs_count.index)), reverse=True)
samples = list(corr_qpcr_count.columns)

# keep only the data for these species (align data)
corr_shared_qpcr = corr_qpcr_count.loc[shared_species, samples]
corr_shared_ngs = corr_ngs_count.loc[shared_species, samples]
assert corr_shared_qpcr.shape == corr_shared_ngs.shape

# use pseudocounts (=1) for the observed data for species detected by HT-qPCR (reference) 
not_observed = (corr_shared_ngs == 0) & (corr_shared_qpcr != 0)
corr_shared_ngs[not_observed] = 1

# remove data points that are only in the observed data (metacal could also remove these automatically) 
no_ref = (corr_shared_ngs != 0) & (corr_shared_qpcr == 0)
corr_shared_ngs[no_ref] = 0
```

In [30]:

```
# copy number correction
CN_bias_raw = pd.DataFrame(index=shared_ngs.index)
CN_bias_raw["Copy_number"] = CN_bias_raw.index.map(cn_bias_dict)
CN_shared_raw = shared_ngs.div(CN_bias_raw["Copy_number"], axis=0).round()

# L. plantarum correction and copy number correction
CN_bias = pd.DataFrame(index=corr_shared_ngs.index)
CN_bias["Copy_number"] = CN_bias.index.map(cn_bias_dict)
CN_shared_ngs = corr_shared_ngs.div(CN_bias["Copy_number"], axis=0).round()

CN_obs_df_raw = CN_shared_raw.copy()
corr_ref_df = corr_shared_qpcr.copy()
corr_obs_df = corr_shared_ngs.copy()
CN_obs_df = CN_shared_ngs.copy()

%R -i corr_ref_df -i corr_obs_df -i CN_obs_df -i CN_obs_df_raw
```

In [31]:

```
%%R
# Bias estimation after copy number correction
reference <- ref_df %>% as("matrix")
CN_raw_observed  <- CN_obs_df_raw %>% as("matrix")

stopifnot(identical(dimnames(observed), dimnames(reference)))

CN_raw_bias_fit <- estimate_bias(
  CN_raw_observed,
  reference, 
  margin = 2, # samples as columns
  boot = TRUE
)

CN_raw_bias_fit.summary <- summary(CN_raw_bias_fit)
CN_raw_bias_table <- CN_raw_bias_fit.summary$coefficients
```

In [32]:

```
%%R
# Bias estimation after L. plantarum correction
corr_reference <- corr_ref_df %>% as("matrix")
corr_observed  <- corr_obs_df %>% as("matrix")

stopifnot(identical(dimnames(observed), dimnames(reference)))

corr_bias_fit <- estimate_bias(
  corr_observed,
  corr_reference, 
  margin = 2, # samples as columns
  boot = TRUE
)

corr_bias_fit.summary <- summary(corr_bias_fit)
corr_bias_table <- corr_bias_fit.summary$coefficients
```

In [33]:

```
%%R
# Bias estimation after L. plantarum correction and copy number correction
corr_reference <- corr_ref_df %>% as("matrix")
CN_observed  <- CN_obs_df %>% as("matrix")

stopifnot(identical(dimnames(observed), dimnames(reference)))

CN_bias_fit <- estimate_bias(
  CN_observed,
  corr_reference, 
  margin = 2, # samples as columns
  boot = TRUE
)

CN_bias_fit.summary <- summary(CN_bias_fit)
CN_bias_table <- CN_bias_fit.summary$coefficients
```

In [34]:

```
%R -o CN_raw_bias_table -o corr_bias_table -o CN_bias_table
```

In [35]:

```
# Calculate the error bars for the plots Fig 4 B, C
corr_biasdf = corr_bias_table.copy()
corr_biasdf["errorbar_min"] = corr_biasdf["estimate"] / (corr_biasdf["gm_se"] ** 2)
corr_biasdf["errorbar_max"] = corr_biasdf["estimate"] * (corr_biasdf["gm_se"] ** 2)

CN_biasdf = CN_bias_table.copy()
CN_biasdf["errorbar_min"] = CN_biasdf["estimate"] / (CN_biasdf["gm_se"] ** 2)
CN_biasdf["errorbar_max"] = CN_biasdf["estimate"] * (CN_biasdf["gm_se"] ** 2)

CN_raw_bias_table
CN_biasdf_raw = CN_raw_bias_table.copy()
CN_biasdf_raw["errorbar_min"] = CN_biasdf_raw["estimate"] / (CN_biasdf_raw["gm_se"] ** 2)
CN_biasdf_raw["errorbar_max"] = CN_biasdf_raw["estimate"] * (CN_biasdf_raw["gm_se"] ** 2)
```

In [36]:

```
# Data Fig 4 A)

# 16S rRNA copy number correction
ngs_raw_CN = ngs_count.copy()
ngs_raw_CN["Copy_number"] = ngs_raw_CN.index.map(cn_bias_dict)
ngs_raw_CN = ngs_raw_CN.div(ngs_raw_CN["Copy_number"], axis=0).round()
ngs_raw_CN_rel = mf.calc_rel_abund(ngs_raw_CN.drop(["Copy_number"], axis=1))

corr_ngs_CN = corr_ngs_count.copy()
corr_ngs_CN["Copy_number"] = corr_ngs_CN.index.map(cn_bias_dict)
corr_ngs_CN = corr_ngs_CN.div(corr_ngs_CN["Copy_number"], axis=0).round()
corr_ngs_CN_rel = mf.calc_rel_abund(corr_ngs_CN.drop(["Copy_number"], axis=1))

# Bray-Curtis Dissimilarity calculation for raw and corrected data

raw_data = {
    "R": qpcr_reldna.copy(),
    "A": ngs_reldna.copy(),
    "B": ngs_raw_CN_rel.copy()
    }

corr_data = {
    "R": corr_qpcr_rel.copy(),
    "C": corr_ngs_rel.copy(),
    "D": corr_ngs_CN_rel.copy()
    }

CN_corr_df_r1 = hf.method_comparison_df(raw_data)
CN_corr_df_r2 = hf.method_comparison_df(corr_data)
dissim_to_ref1 = hf.dissimilarity_df(CN_corr_df_r1, methods=["R", "A", "B"])
dissim_to_ref2 = hf.dissimilarity_df(CN_corr_df_r2, methods=["R", "C", "D"])

dissim_to_ref = pd.concat([dissim_to_ref1, dissim_to_ref2])
```

In [37]:

```
# Figure 4
fig = plt.figure(figsize = (11.5, 8))
# Create axes
ax = gridspec.GridSpec(
    ncols=5, nrows=2, figure=fig, hspace=0.25, wspace=0.025,
    width_ratios=[0.2, 1, 0.45, 1.1, 0.2],
    height_ratios=[0.3, 0.7])

f_buffer = fig.add_subplot(ax[0, 1])
f_ax1 = fig.add_subplot(ax[0, 0:2])
f_ax2 = fig.add_subplot(ax[1, 1])
f_sep = fig.add_subplot(ax[0:,2])
f_ax3 = fig.add_subplot(ax[0:,3])
f_ax4 = fig.add_subplot(ax[0:,4])

f_sep.axis('off')
f_buffer.axis('off')

#colors = sns.color_palette("colorblind", 2)*2
colors = ["grey", "black", "deepskyblue", "darkblue"]
# Plot A
pf.dissimilarity_plot(dissim_to_ref, f_ax1, colors)

# Plot B
#colors = ("black", sns.color_palette("colorblind", 1)[0])
colors = ["grey", "deepskyblue"]
pf.bias_plot(biasdf, corr_biasdf, ax=f_ax2, colors=colors, markers=["o", "^"])
f_ax2.set_xticks([0.1, 0.3, 1, 3])
f_ax2.set_xticklabels([0.1, 0.3, 1, 3])

handles, labels = f_ax2.get_legend_handles_labels()
f_ax2.legend(
        handles, ["raw", "corr."],
        loc='upper left', frameon=False, markerscale=1,
        columnspacing=0.1, labelspacing=0.1,
        handletextpad=0.1, prop={"size": 10},
        bbox_to_anchor=(-0.025, 1.0)
        )

ylim = (0.5, 15.5)
f_ax2.set_ylim(ylim)
f_ax2.tick_params(length=0, which="minor")
f_ax2.grid("on", color="white")
f_ax2.set_facecolor('gainsboro')
for spine in f_ax2.spines:
    f_ax2.spines[spine].set_color("white")

#colors = sns.color_palette("colorblind", 2)*2
colors = ["deepskyblue", "darkblue"]
# Plot C
pf.bias_plot(corr_biasdf, CN_biasdf, ax=f_ax3, colors=colors, markers=["^", "^"])
f_ax3.set_xticks([0.1, 0.3, 1, 3])
f_ax3.set_xticklabels([0.1, 0.3, 1, 3])

handles, labels = f_ax3.get_legend_handles_labels()
f_ax3.legend(
        handles, ["corr.", "corr. GCN"],
        loc='upper left', frameon=False, markerscale=1,
        columnspacing=0.1, labelspacing=0.1,
        handletextpad=0.1, prop={"size": 10},
        bbox_to_anchor=(-0.025, 1.01)
        )

ylim = (0.5, 15.5)
f_ax3.set_ylim(ylim)
f_ax3.tick_params(length=0, which="minor")
f_ax3.grid("on", color="white")
f_ax3.patch.set_facecolor('gainsboro')
for spine in f_ax3.spines:
    f_ax3.spines[spine].set_color("white")

# Plot C copy number barplot
error_order = corr_biasdf.sort_values(["estimate"], ascending=False)["taxon"]
cndf = pd.DataFrame([copy_dict[e] for e in error_order], index=error_order, columns=["16S CN"])
sns.barplot(x="16S CN",y=cndf.index, data=cndf, ax=f_ax4, color="black")
f_ax4.grid('on', which="both", axis="x", color="black")
f_ax4.set_xlim(0, 9.5)
f_ax4.set_xticks([2, 4, 6, 8])
f_ax4.set_yticklabels([])
f_ax4.tick_params(axis="y", length=0)
f_ax4.set_xlabel("")
f_ax4.set_ylabel("")
f_ax4.set_title("Avg.\ncopy number", horizontalalignment='center')
f_ax4.spines['top'].set_visible(False)
f_ax4.spines['right'].set_visible(False)
f_ax4.spines['bottom'].set_visible(False)
f_ax4.tick_params(axis="x", width=0, pad=-0.5)

# Add subplot labels
f_ax1.text(
    -0.425, 1., 'A', fontsize=22, horizontalalignment='center',
     verticalalignment='bottom', transform=f_buffer.transAxes)
f_ax2.text(
    -0.425, 1., 'B', fontsize=22, horizontalalignment='center',
     verticalalignment='bottom', transform=f_ax2.transAxes)
f_ax3.text(
    -0.3, 1., 'C', fontsize=22, horizontalalignment='center',
     verticalalignment='bottom', transform=f_ax3.transAxes)

save_to = os.path.join(figures_dir, "Figure_4.png")
plt.savefig(save_to, dpi=600, bbox_inches='tight')
```

##### Figure 4: Bias estimates for raw and corrected data sets¶

A) Bray-Curtis dissimilarity of relative abundance data of 16S rRNA gene amplicon sequencing (NGS) to HT-qPCR relative abundance data (reference). Depicted are the dissimilarities for the raw and the corrected *L. plantarum* group data sets without and with gene copy number normalization (GCN) for the 16S rRNA gene.
B) Bias point estimates ± two geometric standard errors before and after the correction of the assignments for the *L. plantarum* group species.
C) Bias point estimates ± two geometric standard errors after the correction of the assignments for the *L. plantarum* group species, with and without 16S rRNA gene copy number correction.

#### Bias after correction for *L*. *plantarum* group species¶

- The estimated bias for *L*. *plantarum* after the *L*. *plantarum* group correction is significantly lower than without the correction.
- 16S rRNA gene copy number correction has only a minor influence on the estimated bias.

In [38]:

```
cmap = sns.color_palette("Paired", 12) + sns.color_palette("colorblind")[4::]

# prepare grid
fig = plt.figure(figsize = (18, 14))
ax = gridspec.GridSpec(
    ncols=6, nrows=6, figure=fig, wspace=0.0, 
    hspace=0.5, width_ratios=[0.5, 2, 0.4, 0.4, 0.4, 0.4])

f_ax1 = fig.add_subplot(ax[0:, 0])
f_ax2 = fig.add_subplot(ax[0:, 1])
f_ax3 = fig.add_subplot(ax[0:, 2])
f_ax3.axis("off")

methods=["R", "D"]
refdict = {"R": "qPCR", "D": "NGS"}

search = "[{}]".format("".join(methods))
linkdf = CN_corr_df_r2.filter(regex=search, axis=1).T

# Draw plot and get leaves of linkage
ax, leaves = pf.linkage_plot(linkdf, f_ax1, corr_shared_species)
barplot_data = pf.sort_barplots(linkdf, leaves, corr_shared_species)
pf.draw_linked_barplots(barplot_data, f_ax2, f_ax3, refdict, cmap)

save_to = os.path.join(figures_dir, "Figure_5.png")
plt.savefig(save_to, dpi=600, bbox_inches='tight')
```

##### Figure 5: Comparison of HT-qPCR and 16S rRNA gene amplicon sequencing (NGS) data with corrections for L. plantarum group species and 16S rRNA gene copy normalization.¶

Relative species compositions of the samples measured by HT-qPCR and NGS The samples are sorted and clustered according the UPGMA linkage based on the Bray-Curtis dissimilarity. The relative abundance of the 15 species/groups detected by both methods are depicted with the species name, the other taxa were summarized as other species.

---
