# Supplementary figures and images for: High-throughput qPCR and 16S rRNA gene amplicon sequencing as complementary methods for the investigation of the cheese microbiota
Source: BMC Microbiol. 2022 Feb 7;22:48. doi: 10.1186/s12866-022-02451-y (PMC8819918; doi:10.1186/s12866-022-02451-y)

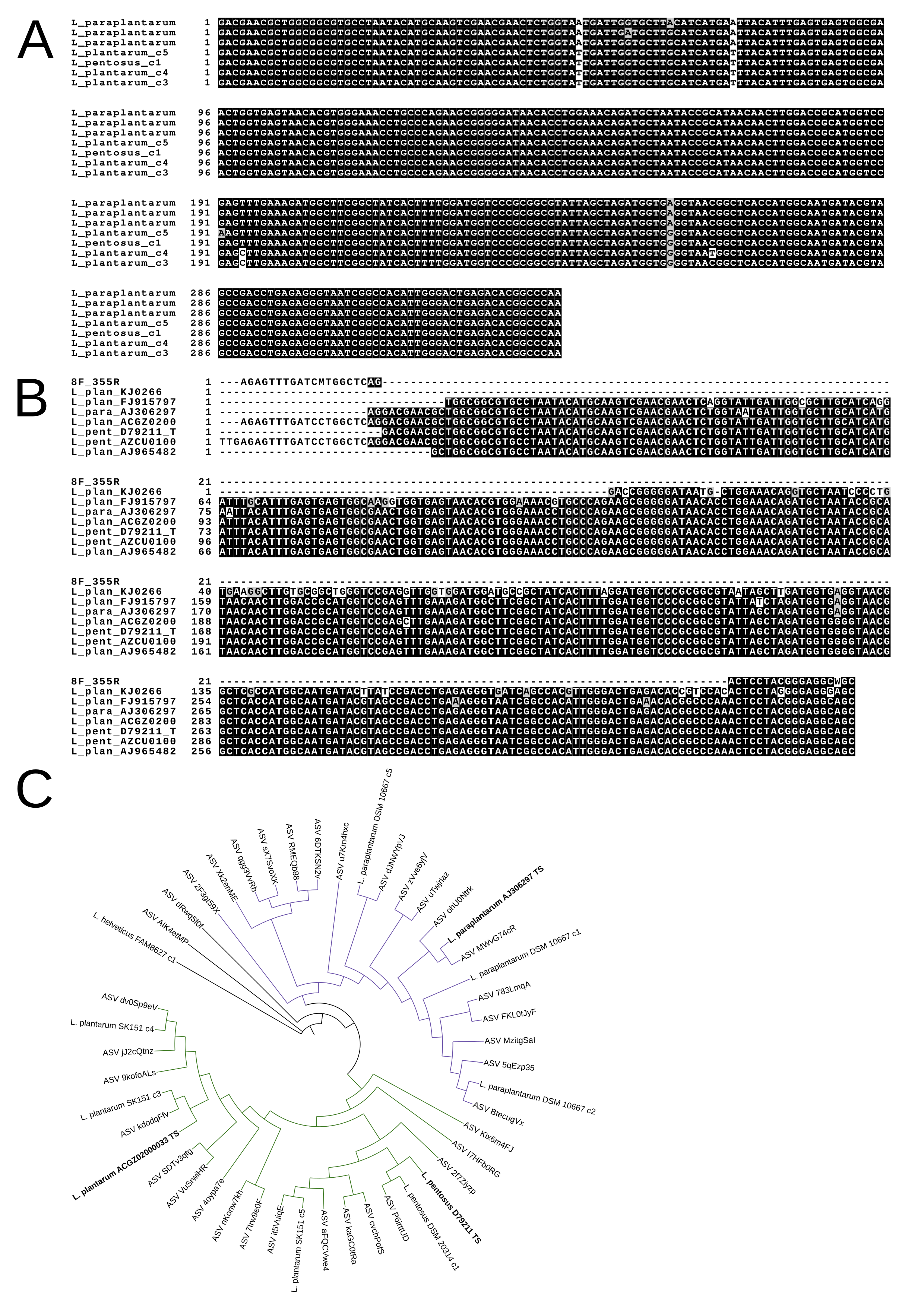

Supplement: Supplementary file 1 — Additional file 1. [file 12866_2022_2451_MOESM1_ESM.zip › htqpcr_ngs_data-main/Figures/Supplementary_Figure_S1.png]

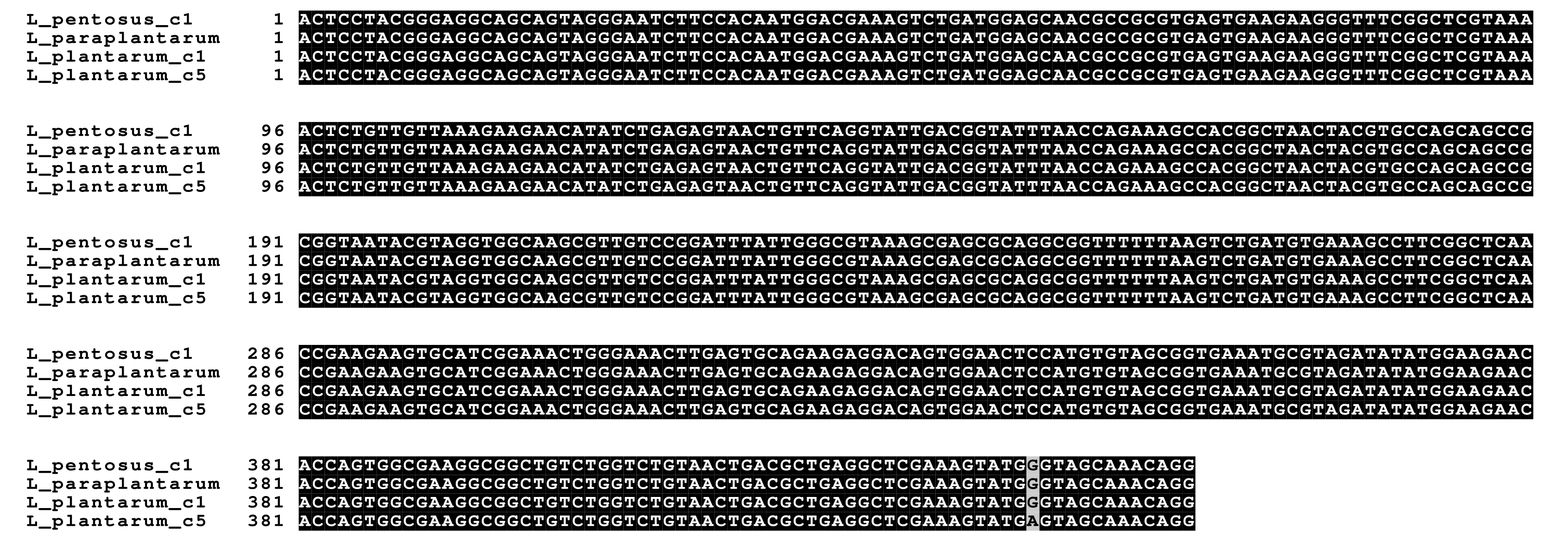

Supplement: Supplementary file 1 — Additional file 1. [file 12866_2022_2451_MOESM1_ESM.zip › htqpcr_ngs_data-main/Figures/Supplementary_Figure_S2.png]

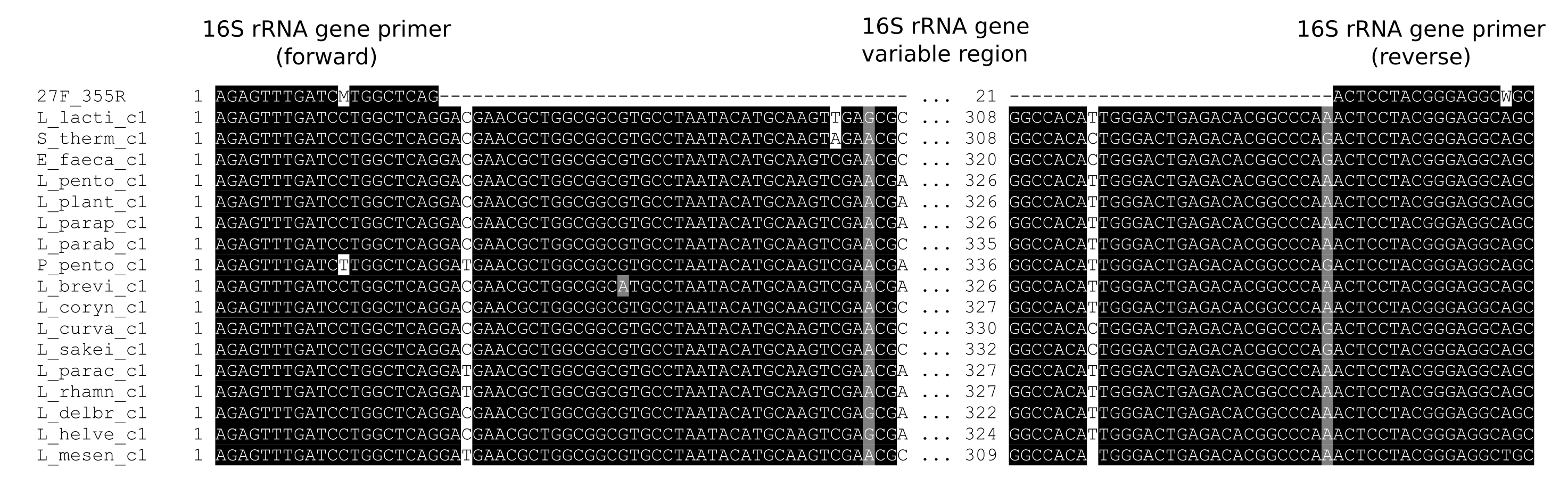

Supplement: Supplementary file 1 — Additional file 1. [file 12866_2022_2451_MOESM1_ESM.zip › htqpcr_ngs_data-main/Figures/Supplementary_Figure_S3.png]
