# Supplementary material for: High-throughput qPCR and 16S rRNA gene amplicon sequencing as complementary methods for the investigation of the cheese microbiota
Source: BMC Microbiol. 2022 Feb 7;22:48. doi: 10.1186/s12866-022-02451-y (PMC8819918; doi:10.1186/s12866-022-02451-y)
Supplement: Supplementary file 3 — Additional file 3. [file 12866_2022_2451_MOESM3_ESM.pdf]

Supplementary figures

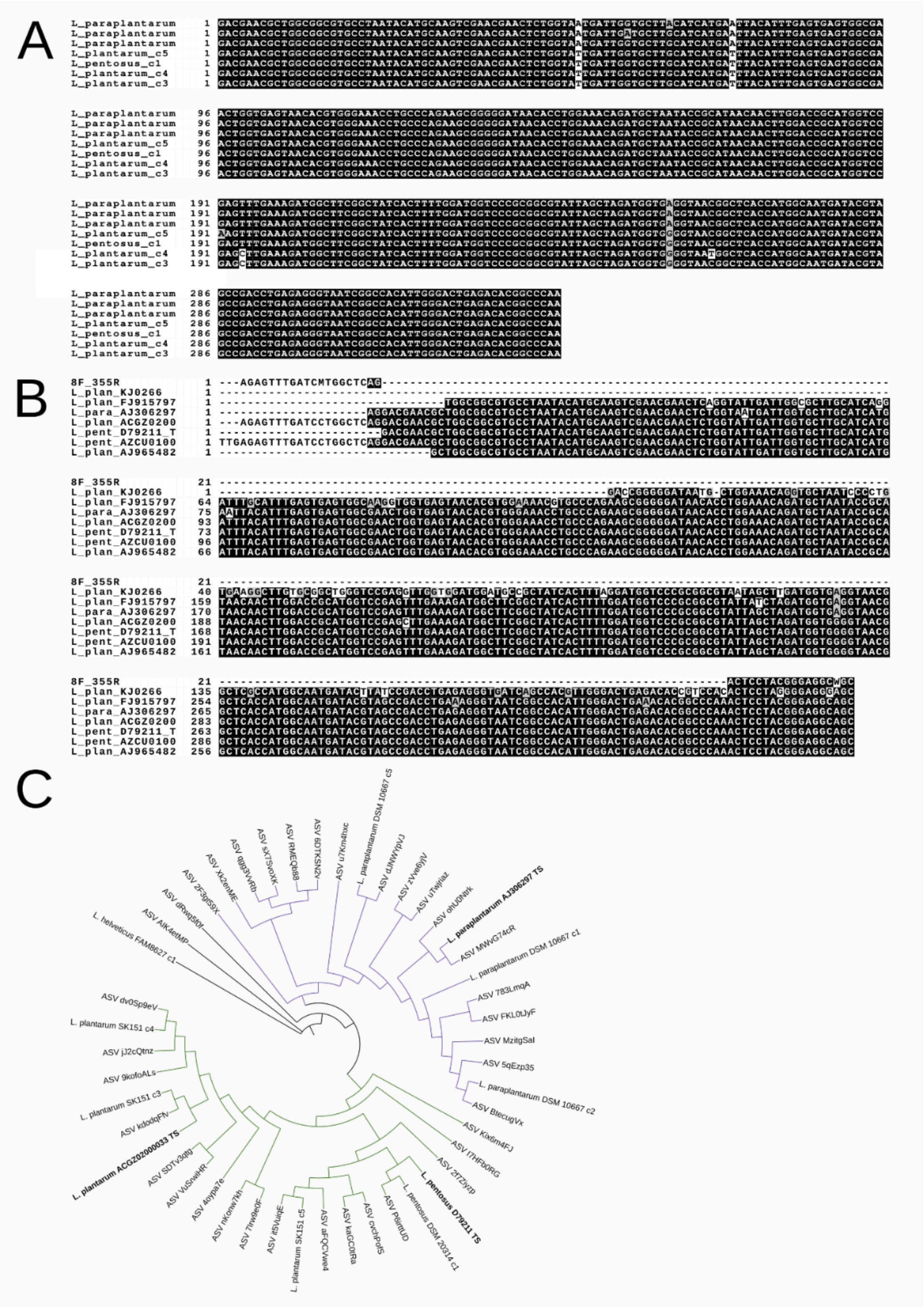

Supplementary Figure S1: Analysis of the potential to differentiate *Lactiplantibacillus plantarum* group species based on V1–V2 variable regions of the 16S rRNA gene
A) Alignment of the V1–V2 16S rRNA gene region of the representative genomes of *Lactiplantibacillus plantarum*, *L. paraplantarum* and *L. pentosus*. B) Alignment of the DAIRYdb 16S rRNA gene reference sequences. C) The best-scoring maximum likelihood tree from alignments of the V1–V2 16S rRNA gene sequences including the representative genomes, the DAIRYdb reference sequences, and the ASVs for *L. plantarum*, *L. paraplantarum* and *L. pentosus*.

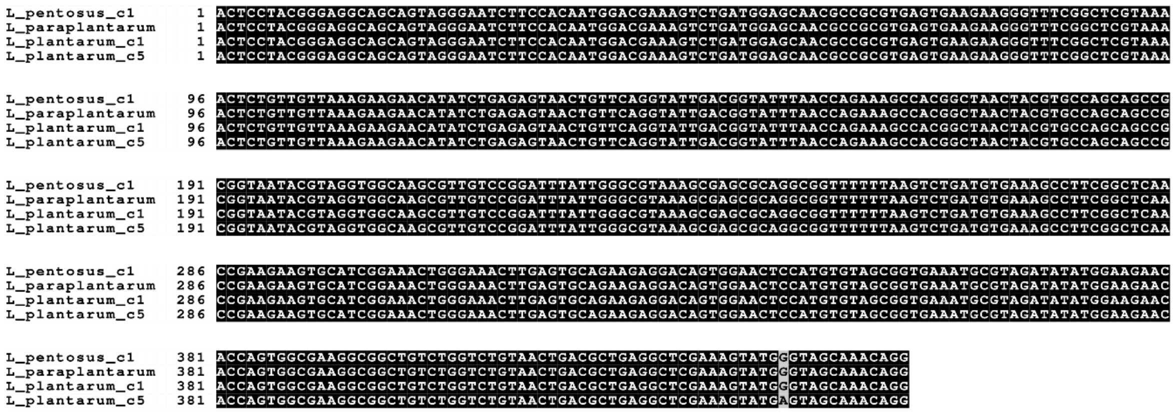

Supplementary Figure S2: ***Lactiplantibacillus plantarum*** **group discrimination of 16S rRNA gene amplicon sequencing targeting the V3–V4 region**

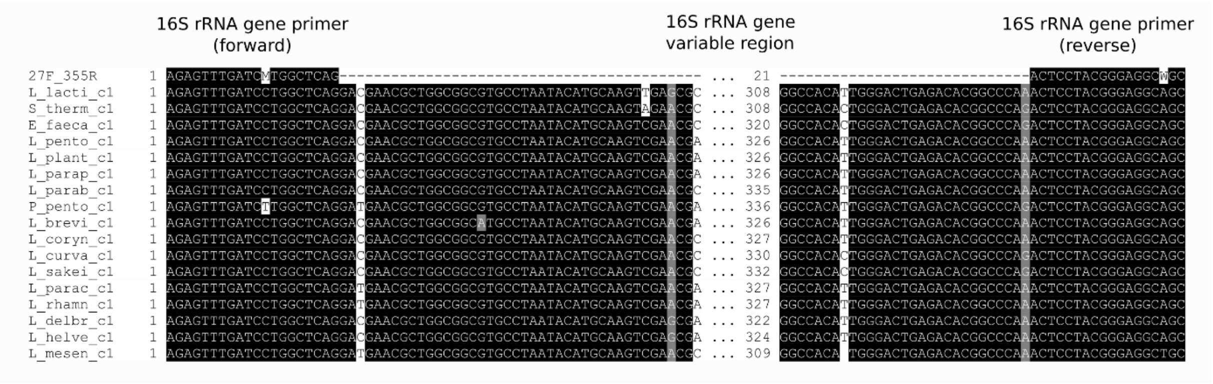

Supplementary Figure S3: Alignment of the 16S rRNA gene primer binding regions
